# Improving Evolutionary Models for Mitochondrial Protein Data with Site-Class Specific Amino Acid Exchangeability Matrices

**DOI:** 10.1371/journal.pone.0055816

**Published:** 2013-01-31

**Authors:** Katherine A. Dunn, Wenyi Jiang, Christopher Field, Joseph P. Bielawski

**Affiliations:** 1 Department of Biology, Dalhousie University, Halifax, Nova Scotia, Canada; 2 Department of Mathematics and Statistics, Dalhousie University, Halifax, Nova Scotia, Canada; University of Lausanne, Switzerland

## Abstract

Adequate modeling of mitochondrial sequence evolution is an essential component of mitochondrial phylogenomics (comparative mitogenomics). There is wide recognition within the field that lineage-specific aspects of mitochondrial evolution should be accommodated through lineage-specific amino-acid exchangeability matrices (*e.g*., mtMam for mammalian data). However, such a matrix must be applied to all sites and this implies that all sites are subject to the same, or largely similar, evolutionary constraints. This assumption is unjustified. Indeed, substantial differences are expected to arise from three-dimensional structures that impose different physiochemical environments on individual amino acid residues. The objectives of this paper are (1) to investigate the extent to which amino acid evolution varies among sites of mitochondrial proteins, and (2) to assess the potential benefits of explicitly modeling such variability. To achieve this, we developed a novel method for partitioning sites based on amino acid physiochemical properties. We apply this method to two datasets derived from complete mitochondrial genomes of mammals and fish, and use maximum likelihood to estimate amino acid exchangeabilities for the different groups of sites. Using this approach we identified large groups of sites evolving under unique physiochemical constraints. Estimates of amino acid exchangeabilities differed significantly among such groups. Moreover, we found that joint estimates of amino acid exchangeabilities do not adequately represent the natural variability in evolutionary processes among sites of mitochondrial proteins. Significant improvements in likelihood are obtained when the new matrices are employed. We also find that maximum likelihood estimates of branch lengths can be strongly impacted. We provide sets of matrices suitable for groups of sites subject to similar physiochemical constraints, and discuss how they might be used to analyze real data. We also discuss how the general approach might be employed to improve a variety of mitogenomic-based research activities.

## Introduction

High throughput sequencing technology has led to renewed interest in mitochondrial gene sequences as a means of inferring species relationships. The greatly expanded sequencing capacity makes feasible phylogenetic inference from complete mitochondrial genomes, or from the complete set of mitochondrially-encoded proteins. Given that those data can be readily obtained from most species, and that the genomes are typically non-recombining and fast-evolving, mitochondrial-genome based studies currently represent one of the most frequent forms of phylogenomics (*e.g.*, [1]–[3]). For the same reasons, mitochondrial genomes are now being widely used for molecular dating of divergence events (*e.g*., [4]–[6]). However, effective use of mitochondrial sequences for these tasks does pose some challenges; with the most common one being loss of signal due to saturation of nucleotide substitutions among the more divergent sequences [4]. Hence, deep-level mitochondrial phylogenomics ordinarily involves analysis of amino acid variability.

Adequate modeling of the amino acid substitution process is critical to inferring a phylogeny and to estimating divergence dates. The most widely used approach is to accommodate variability in replacement rates between different amino acids by using empirical estimates derived from a large database of proteins (e.g., [7], [8]) and to model among sites variability in evolutionary rate by using a parametric distribution such as gamma [9], [10]. Empirical estimates of the 189 amino acid replacement rates are used because it is difficult to reliably estimate so many parameters from a single dataset, as well as being computationally very costly. Recent attempts to improve models of protein evolution were motivated by variability among sites in the “pattern” of amino acid replacement rates (in addition to among sites rate variation), presumably arising from site-specific structural interactions and functional constraints (*e.g*., [11]–[13]). Several authors have modeled such variation by permitting the equilibrium frequencies of the 20 amino acids to vary among sites (e.g., [14]–[17]). Additional improvements were achieved by permitting exchangeability parameters (*sc.* [7] and *q.v*. methods), as well as equilibrium frequencies and evolutionary rates, to vary among sites [18]–[19]. While such models have not yet been widely adopted in phylogenomics, it appears that inadequate modeling of process variability among-sites can be responsible for phylogenetic artifacts such as long-branch attraction [16], [17], [19], [20].

Le et al. [18] showed that a single matrix of amino acid exchangeabilities was insufficient to fully represent the complexity of among site variation in solvent exposure, secondary and tertiary structure, and functional constraints. They achieved highly significant improvements in fits to real data by constructing mixture models that combine several different matrices of amino acid exchangeabilities. Their matrices were estimated either for pre-defined structural categories (based on solvent exposure or secondary structure) or for partitions derived from an unsupervised learning technique. The use of unsupervised matrices tended to outperform matrices derived from structural categories, suggesting that the pre-defined categories were not sufficient to capture the full extent of among-sites evolutionary variation. However, because their models mix for both the overall rate of evolution and the amino acid exchangeabilities they incur a substantial computational liability. To reduce this computation burden, Le et al. [19] developed simpler models that extend among-sites mixture model for rates (*e.g*., [9]) so that each rate class has a unique exchangeability matrix. They [19] use a supervised and semi-supervised procedure to estimate rate-class specific exchangeability matrices. Their results corroborate the earlier finding [18] that substantial improvements can be obtained by permitting exchangeabilities to vary among sites.

The empirical exchangeability matrices of [18] and [19], although obtained by using a very large alignment database, are intended for use with “generalized” globular proteins. It is well known that such matrices will not be suitable for certain protein groups (*e.g*., transmembrane or mitochondrial proteins) or domains of life (*e.g*., viruses). Indeed, for mitochondrial proteins it seems that unique exchangeability matrices are best estimated for specific lineages (*e.g*., mtMam [21], mtArt [22], mtPan [23], mtZoa [24]). We predict that even these linage-specific mitochondrial matrices, which are applied as a single matrix to all sites, might be insufficient to fully represent the complexity of mitochondrial amino acid evolution.

The focus of this paper is to investigate the extent to which the process of amino acid evolution varies among sites of mitochondrial proteins. We formally present (1) a new unsupervised learning method for partitioning sites based on amino acid physiochemical properties, and (2) sets of empirical exchangeability matrices derived from partitions identified by the new method. We apply our new method to two large datasets derived from complete mitochondrial genomes of mammals and fish. The significance of these results are assessed via noise-analysis and cross-validation procedures. Lastly, we discuss how several different mitogenomic-based research activities could be improved by better modeling of the natural variability in evolutionary processes among sites of mitochondrial proteins.

## Results and Discussion

Application of a single rate matrix, such as mtMam [21], to all mitochondrial protein-coding genes implies that all those sites are subject to the same evolutionary constraints. This assumption is too simplistic; proteins fold into three-dimensional structures that impose different chemical environments on individual amino acid residues and thereby impose different evolutionary constraints on the acceptability of different amino acids. Here, we assume that sites belong to one of several groups subject to different evolutionary constraints, leading to similarities in the physiochemical properties only among the predominant amino acids within the same group. We present a novel method for transforming each site within a multiple sequences alignment (MSA) according to the physiochemical properties of its amino acids and then clustering them into discrete groups. Rather than fix the number of groups *a priori*, gap-statistics are used to determine the ideal number of groups for the data in hand. The original amino acid states corresponding to each site within a group are used to estimate a matrix of instantaneous substitution rates specific to that group. Both a noise-analysis and cross-validation are employed to evaluate the significance of the differences between rate matrices. The methods are applied to two large mitochondrial datasets.

### A novel method for transformation and clustering of sites according to the physiochemical properties of the amino acids

**Figure 1:**
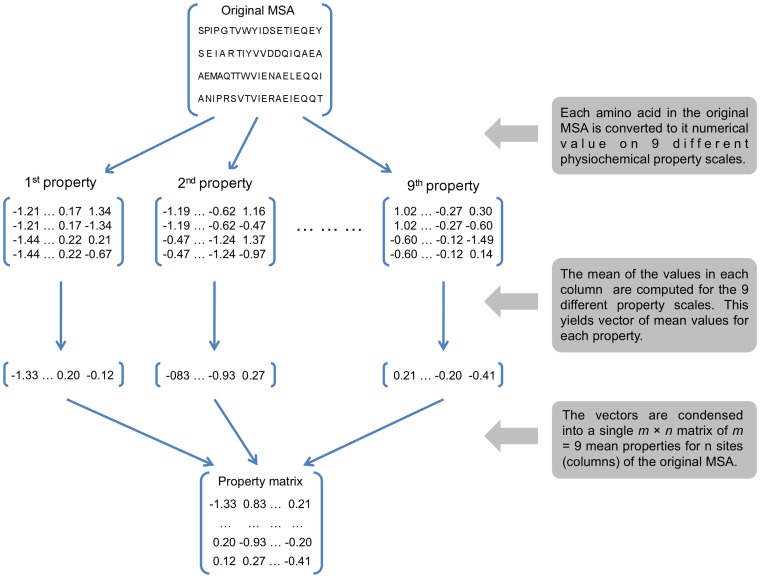
Schematic overview of the transformation of a matrix of sequence data to a physiochemical property matrix. The original matrix is a multi-sequence alignment (MSA) of amino acid sequences having *n* columns (sites). Each amino acid is converted to a numerical value on a particular physiochemical scale. To capture the complexity of the physiochemical effects, we employed *m* = 9 different physiochemical scales in this study (see Table 1). Thus nine different transformations of the MSA were carried out. The data are then condensed into a single *n*×*m* matrix by computing the mean for each property at each site and constructing a column vector having *m* = 9 different mean values for each site. Thus the final matrix has *n* alignment sites and *m* physiochemical properties.

Starting with a MSA of amino acids, in the standard alphabetic format, the first step is to transform each site (column) in the MSA into a vector of numerical information representing the physiochemical properties of the amino acids at that site. This is done by replacing the alphabetic designation of each amino acid state with its corresponding value on a particular physiochemical scale. At a given site, each amino acid present is replaced by the *m* different measures of physiochemical properties for that amino acid. The mean of each of the *m* measures is computed for the column vector corresponding to each site in the MSA. This yields, for each site, *m* different mean values for the *m* physiochemical properties, and these are assembled into a new *n*×*m* matrix where *n* is the number of sites in the MSA (see Figure 1 for an overview).

There are well over 100 different scales for measuring the physiochemical property of an amino acid. Moreover, many of these are not independent, as they represent alternative measures of the same scale (*e.g.*, there are 4 measures of hydrophobicity in the APDbase [25] and 34 in the AAindex database [26]). Kidera et al [27] used multivariate statistical methods to reduce a set of 188 different measures of physiochemical property to a set of nine, largely orthogonal, property scales. We employed these nine measures to transform our data as described above. Hence *m* = 9 hereafter, with one property measuring bulk (P1), two measuring hydrophobicity in free amino acids (P2, P3), one measuring hydrophobicity in proteins (P4), two measuring the preference of an amino acid for *β*-structures (P5, P6), one measuring preference for *α*-helices (P7), and two measuring a preference for forming a bend-structure (P8, P9). The value of each of the 20 amino acids on each of the above 9 measurement scales is provided in table 1.

**Table 1:** The value of each amino acid according to 9 different physiochemical property scales.

Amino acid		P1	P2	P3	P4	P5	P6	P7	P8	P9
Ala	A	−1.44	−0.47	0.11	0.32	−0.51	−0.86	1.35	−1.29	−0.6
Arg	R	1.16	−0.57	−1.52	−1.07	−0.28	−0.13	−0.16	0.28	−0.03
Asn	N	−0.34	−1.25	−0.6	−0.96	−1	−1.19	−0.97	1.19	1.27
Asp	D	−0.54	−0.75	−1.74	−1.07	−1.17	−1.72	−0.06	0.74	1.39
Cys	C	−0.75	0.06	0.63	1.5	0.6	1.14	−0.53	1.18	−0.19
Gln	Q	0.22	−1.24	−0.46	−1.05	0.19	−0.42	0.57	−0.14	−0.12
Glu	E	0.17	−0.62	−1.65	−1.03	−1.74	−1.78	1.96	−1.21	−0.27
Gly	G	−2.16	−1.02	−0.19	−0.03	−0.84	−0.99	−1.72	1.43	1.73
His	H	0.52	−0.46	−0.18	−0.13	−0.56	−0.1	0.59	−0.27	−0.27
Ile	I	0.21	1.37	0.97	1.52	1.91	1.27	0.06	−1.3	−1.49
Leu	L	0.25	1.06	1.01	1.14	0.69	0.02	0.93	−1.36	−1.14
Lys	K	0.68	−0.16	−1.62	−1.76	−0.86	−1.19	0.71	0.4	0.15
Met	M	0.44	0.2	0.72	1	0.45	0.24	1.39	−1.24	−1.29
Phe	F	1.09	1.46	1.24	1.16	0.88	0.48	0.37	−0.46	−0.75
Pro	P	−0.71	0.9	0.21	−0.72	−1.26	0.86	−1.72	1.03	1.98
Ser	S	−1.21	−1.19	−0.33	−0.46	−0.54	0.22	−0.99	0.74	1.02
Thr	T	−0.67	−0.97	0.01	−0.36	0.57	0.86	−0.68	0.11	0.14
Trp	W	2.08	2.06	1.55	0.67	0.61	0.42	0.23	0.83	−0.52
Tyr	Y	1.34	1.16	1.04	−0.07	1.02	1.21	−1.25	0.94	0.3
Val	V	−0.34	0.42	0.77	1.38	1.84	1.66	−0.09	−1.63	−1.32

Physiochemical property scales are from [27]. P1: bulk; P2–P4: hydrophobicity; P5–P6: *β*-structure preference; P7: *α*-helix preference; P8–P9: bend-structure preference.

To identify groups of sites having similar constraints on the physiochemical properties of their amino acids, we apply a K-means clustering algorithm to the *n*×*m* matrix of mean physiochemical properties. Recall that the columns of this matrix are site-specific physiochemical property vectors. To start, these vectors are assigned at random to *k* different groups (clusters). Based on this random assignment, an initial physiochemical-centroid is computed as the point within the group that minimizes the distances of all the sites in that group to the point. The algorithm then iteratively moves the site-specific column vectors among groups until the distances among member data points and a physiochemical-centroid are minimized. Note that after sites have been moved, *k* new centroids are re-calculated; hence, a stopping criterion for the algorithm can be the point when the physiochemical-centroids no longer change. As the application of this algorithm to a random initialization could lead to a local minimum, we apply the algorithm to 1000 different random initial assignments.

We let the signal in the data decide the optimal number of *k* groups by using an approach based on the “gap” method [28]. The gap measures the distance from the within-cluster dispersion to that expected under an appropriate reference null distribution. The error is measured as the pooled within-cluster sum of squares around the cluster means, and the basic idea of the gap statistic is to compare the error measure with its expectation under a null reference distribution for the data. The optimal number of clusters is found at the point where the value of the error measure for *k* falls the farthest below the reference curve. The reference null distribution is an appropriate uniform distribution, which takes the shape of the data into account. We use the " 1-standard-error” rule to select *k*. See the methods section for additional details.

We applied the methods described above to two mitochondrial datasets. The “mammal dataset” is comprised on 12 mitochondrial proteins (3580 MSA sites) from 143 lineages of mammals and is provided in table S1. The “fish dataset” is comprised of 11 mitochondrial proteins (3370 MSA sites) from 75 lineages of fish and is provided in table S1. Further details about these datasets are provided in the methods section as well as methods S1. MSAs for each dataset were transformed into physiochemical property matrices (Mammal dataset: 3580×9; Fish dataset: 3370×9), and are provided as supplementary information (denoted PmatrixS1 and PmatrixS2).

**Figure 2:**
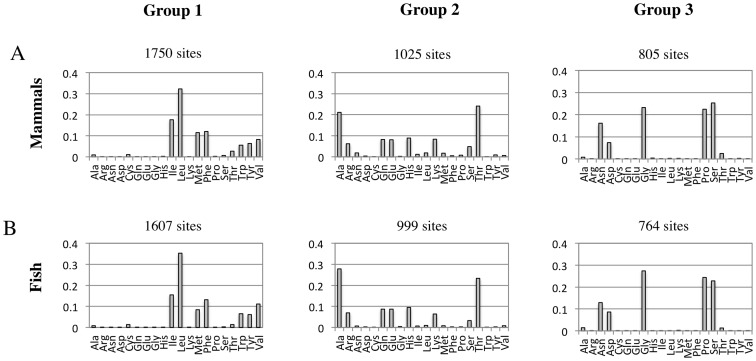
Amino acid composition of groups of sites resolved by K-means clustering on physiochemical properties. The amino acid frequencies in the mammal (**A**) and fish (**B**) datasets differ substantially among the groupings. The mammal dataset is comprised of 3580 MSA sites from 143 mitochondrial genomes and the fish data is comprised of 3370 MSA sites from 75 mitochondrial genomes. Amino acid frequencies are shown for each group of sites.

**Figure 3:**
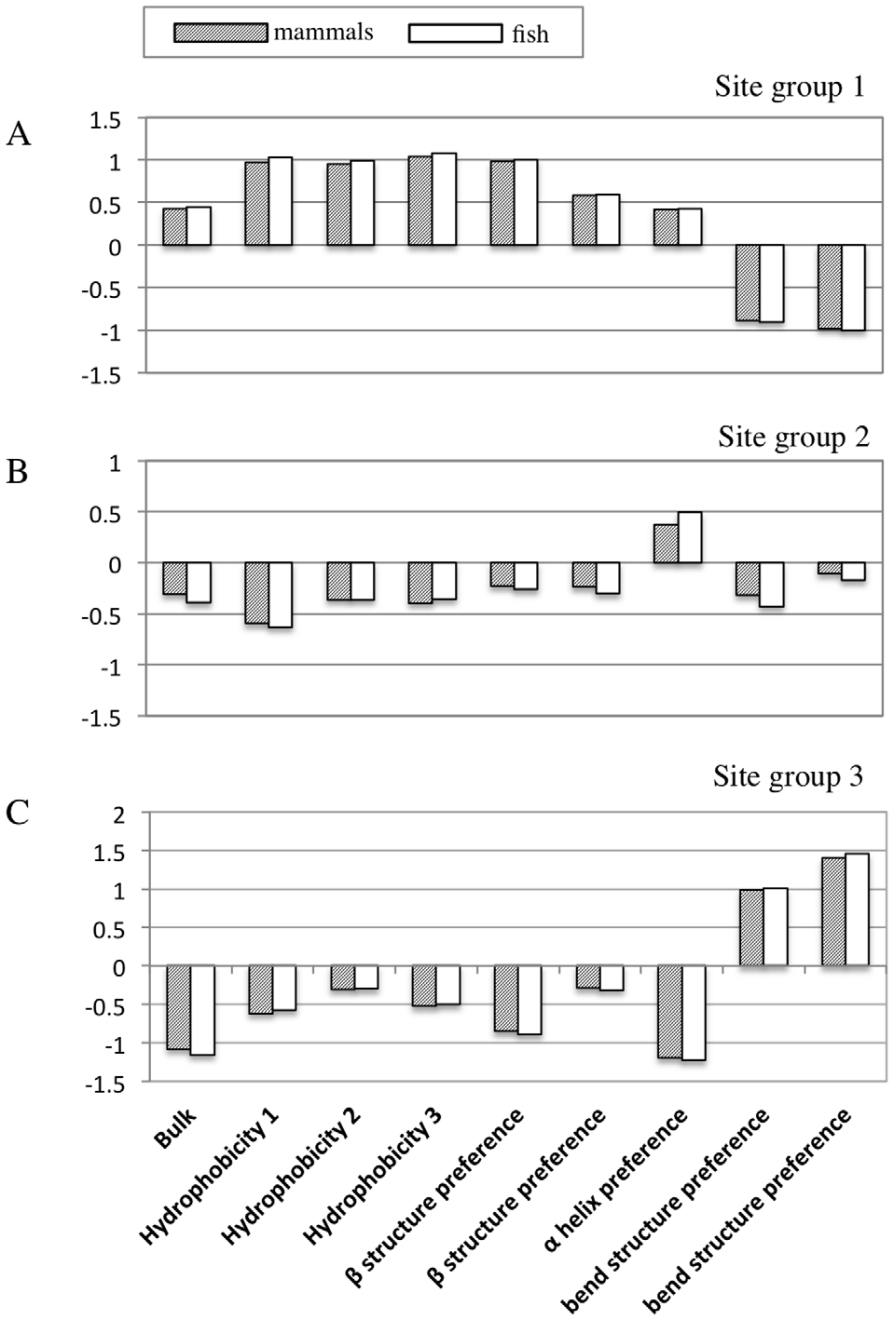
Physiochemical centroids for three groups of sites resolved by K-means clustering. Panel (**A**) shows results for the largest group of sites (1750 for mammals and 1607 for fish). The intermediate group (**B**) was compromised of 1025 sites for mammals and 999 sites for fish. The smallest group (**C**) was compromised of 805 sites for mammals and 764 sites for fish. The physiochemical properties of each of these groups were very similar for mammals and fish.

Analysis of the physiochemical property matrix for the mammal dataset using the “1-standard-error” rule of [28] indicated three groups of sites. Table 2 provides the gap(*k*) and *S_k_* statistics for the clustering under *k* = 2 to *k* = 4 groups of sites. At *k* = 3, the groups contain 1750, 1025 and 805 amino acid sites. Amino acid frequencies within each group are shown in Figure 2. Presumably, these groups represent subsets of sites evolving under unique physiochemical constraints, as they have substantially different empirical frequencies; group 1 (1750 sites) is dominated by leucine and isoleucine, group 2 (1025 sites) is dominated by alanine and threonine, and group 3 (805 sites) is almost completely comprised of just four amino acids (glycine, proline, serine and asparagine). Examination of the centers of the groups suggests the following physiochemical signatures; group 1 amino acids tend to be bulky and hydrophobic, and are amenable to alpha helices and beta structures; group 2 amino acids tend to be more hydrophilic and are amenable to helical structures; group 3 amino acids tend to be less bulky, and disfavor alpha helices in favor of bends. Figure 3 summarizes the pattern of the physiochemical centers of each group of sites. The analysis of the fish-dataset also indicated a *k* of 3, and split the data into 1607, 999, and 764 amino acid sites. The properties of these groups were very similar to the mammal groups of similar size (Figures 2 and 3).

**Table 2:** *Gap(k)* and *S_k_* statistics for the mammal and fish mitochondrial datasets.

Mammal dataset			
Number of partitions	*Gap(k)*	*S_k_*	*Gap(k)-S_k_*
*k* = 2	0.5980	0.0087	0.5893
*k* = 3	0.6371	0.0073	**0.6298**
*k* = 4	0.6209	0.0066	0.6142

Fish dataset			
Number of partitions	*Gap(k)*	*S_k_*	*Gap(k)-S_k_*
*k* = 2	0.5109	0.0087	0.5021
*k* = 3	0.5789	0.0067	**0.5722**
*k* = 4	0.5618	0.0070	0.5548

The gap is a measurement of the difference between the error within a group and its expected value under a reference (null) distribution. *S_k_* is the standard deviation of the log of distance vectors of the reference data for *k* clusters *Gap(k)*. The value of *k* is chosen as the smallest *k* where *Gap(k)* ≥ *Gap(k+1)−S_k+1_* and is shown in bold.

### ML estimation of amino acid exchangeabilities

**Figure 4:**
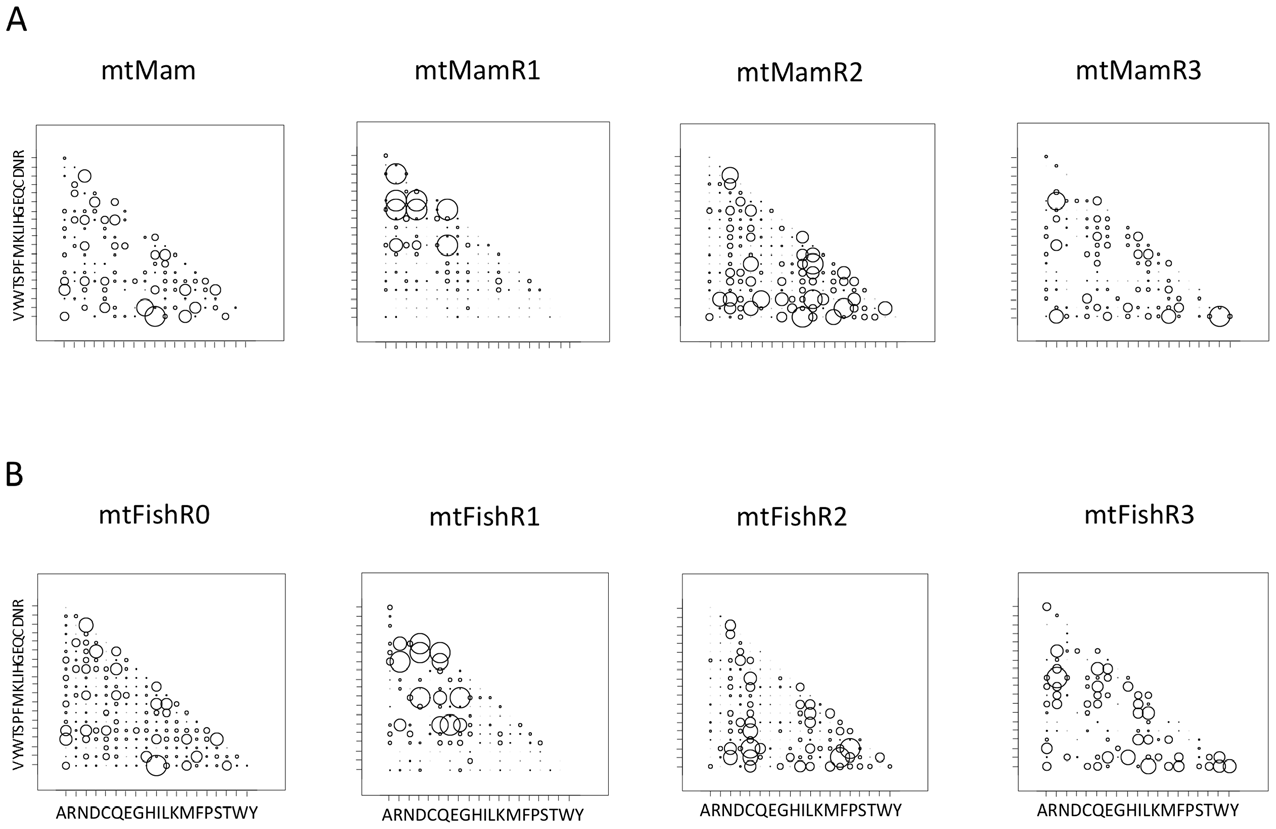
Plots of empirically estimated rate matrices (*R*) for complete and partitioned sets of (A) mammalian and (B) fish mitochondrial sequences. Exchangeability parameters of the rate matrices were estimated by maximum likelihood. These parameters are plotted as circles within a 20×20 matrix, where the diameter of the circle is proportional to parameter value.

If the groups identified above represent sites subject to different physiochemical constraints, then the dynamics of amino acid evolution should differ among those groups. To investigate this for each group of sites identified, we estimate a matrix of amino acid exchangeabilities (*R*) for the original protein sequences corresponding to each group of sites identified by K-means clustering on their physiochemical properties. The parameters of the *R* matrix, along with branch lengths, are estimated by maximum likelihood using the codeml program of PAML [9] under a fixed tree topology. Here, two different methods are used to estimate the *R* matrices, with each method initiated from several different sets of values for the amino acid exchangeabilities (see the methods section for additional details). Different methods sometimes yielded different *R* matrices. In such cases, the matrix having the highest likelihood score is taken as the best estimate of *R*. Bubble-plots are used to visualize the *R* matrices, where the size of a bubble is proportional to the inferred substitution rate and is comparable across different matrices (Figure 4).

First we estimated an *R* matrix jointly for all sites in the mammal dataset. This matrix is similar to the published mtMam matrix in that it also implies that all sites are subject to the same evolutionary constraints. Our estimate of such a matrix (denoted as mtMamR0) was very similar to mtMam (see Figure S1), which is not surprising given that our sample of data covers the breadth of mammalian diversity sampled by [21]. Our sample differs by including more lineages, which does not appear important to the estimate of *R* in this case. All subsequent comparisons will be made with the previously published matrix, mtMam.

Figure 4A presents the *R* matrix for mtMam, and for the three sets of sites grouped according to their physiochemical properties. Hereafter the *R* matrix for the large group (1750 sites) will be referred to as mtMamR1, the *R* matrix for the medium group (1025 sites) as mtMamR2, and the *R* matrix for the small group (805) as mtMamR3. Each matrix is provided as supporting information (RmatricesS1). Figure 4A clearly illustrates that substantial differences exist both between the group-specific *R* matrices and mtMam, suggesting that such a joint *R* matrix is insufficient to represent the site-specific physiochemical constraints that impact substitution dynamics. Likelihood scores presented in table 3 support this interpretation. For example, mtMamR1 is a substantially better fit to group-1 sites than mtMamR2, mtMamR3, or mtMam. However, mtMam did outperform mtMamR2 and mtMamR3 for group-1 sites. Indeed, this pattern of results was also observed for group-2 and group-3, suggesting that mtMam might be the best alternative *R* matrix in the absence of a group-specific *R* matrix. This finding is not surprising because mtMam represents an aggregation of information about amino acid exchangeabilities over all three groups.

**Table 3:** Likelihood of full dataset and three partitions based on a rate matrix estimated from the complete data (R0) and three partition-specific rate matrices (R1, R2 and R3).

Mammal dataset (3580 sites)				
			Subset of data	
			(no. of sites)	
		Group 1	Group 2	Group 3
*R* matrix	Full data	(1750)	(1025)	(805)
mtMamR0	−216607.19	−119282.36	−76399.18	−35438.41
mtMamR1	−333153.29	−106238.09	−109937.12	−46677.37
mtMamR2	−256105.56	−137415.88	−63583.77	−40892.74
mtMamR3	−274537.25	−140685.03	−96717.46	−28146.37

Fish dataset (3370 sites)				
			Subset of data	
			(no. of sites)	
		Group 1	Group 2	Group 3
*R* matrix	Full data	(1607)	(999)	(764)
mtFishR0	−110263.36	−58780.87	−39944.41	−19350.11
mtFishR1	−177238.67	−52027.87	−51906.35	−24749.97
mtFishR2	−137331.88	−67316.29	−32627.99	−23242.22
mtFishR3	−148474.98	−74164.24	−47841.31	−15173.94

The best likelihood score is shown in bold.

Results for the fish dataset were very similar to those obtained for the mammals (Figure 4B). In this case there is no published fish-specific *R* matrix, so we provide ours as supporting information (RmatricesS2), and hereafter refer to this matrix as mtFishR0. The mtFishR0 is estimated under the tree topology estimated from the mitochondrial data in hand. However, the phylogenetic relationships for the fish lineages are somewhat more controversial [29]. To investigate the impact of this uncertainty on the estimate of mtFish matrices we estimated the exchangeabilities under an alternative topology derived from published analyses of morphological characters [30]. The resulting *R* matrices are similar but not identical (see RmatricesS2). Since the impact of topology was small, we present the results inferred under the topology estimated from the data in hand (Figure 4B). As with the mammalian dataset, the group-specific *R* matrices provided a substantially better fit than the alternatives, with the matrix mtFishR0 always the second best likelihood score (table 3). Matrices mtFishR1 (1607 sites), mtFishR2 (999 sites) and mtFishR3 (764 sites) are provided on-line as supporting information (RmatricesS2).

The above analyses were performed without requiring consistency among branch lengths estimated for the three groups of sites. An alternative approach is to constrain the optimized branch lengths so that they are proportional among groups. To achieve this we fit via ML a branch-length scale parameter to each data partition relative to the branch lengths estimated under a joint *R* matrix (*R*0). The effect is that the branch lengths at a site will be proportionally lengthened or shortened according to the value of *s* for the group to which a site belongs; i.e., site-group 1 has 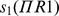; site-group 2 has 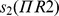; and site-group 3 has 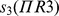. Results were similar to those obtained previously. In both the mammalian and fish datasets, the group-specific *R* matrices provided a substantially better fit than the alternatives (table S2). Because one of our objectives is to explore how branch lengths might be differently impacted within a partition, we chose to base our subsequent analyses on the unconstrained approach to branch length estimation. We note, however, that considerable computational savings can be achieved with the constrained estimation approach.

### Noise analysis

We have assumed that differences in selective constraints on physiochemical properties leads to different evolutionary dynamics within different groups of sites. However, lacking any biological basis for the observed variation in amino acid frequencies among sites, our separation of sites into groups could reflect statistical noise. In a case where groups were separated completely by noise, the group-specific *R* matrices are expected to show some variation in pattern. To investigate if the observed *R* matrices have more structure than expected we carried out a noise analysis.

The original MSA can be randomly partitioned into three groups of size equal to those inferred by clustering the physiochemical property matrix. This random partitioning was repeated 50 times for a given dataset. An *R* matrix was then estimated from every random partition, providing a baseline of results based solely on “noise”. In the methods section we describe a simple measure of the distance between two matrices, and we use this to measure the distance of each random matrix to a reference matrix (in this case, the *R* matrix estimated jointly for all sites in the data). We also measure the distance of non-random *R* matrices (*e.g*., mtMamR1, mtMamR2 and mtMamR3) to a reference matrix (*e.g*., mtMamR0). Results (table 4) allow the use of the one-sample *t*-test to assess if the distance of the non-random matrix from the reference matrix is consistent with random partitioning of sites into groups. In both the mammal and fish datasets the distance of the group-specific matrices is significantly larger than expected if the data had been separated completely by noise arising by sampling errors (table 4).

**Table 4:** Tests of the hypothesis that group-specific *R* matrices have more structure than expected by chance (noise analysis).

Mammal dataset			
	*i* = 1 (large)	*i* = 2 (medium)	*i* = 3 (small)
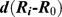	1.7299	1.1334	1.3230
Mean 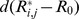	0.1412	0.2140	0.2982
SD 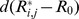	0.0177	0.0295	0.2094
*t-*statistic	−55.24	−50.67	−8.85
*p-*value	5.25e-13	1.40e-12	5.0e-06
*p*_Bonf_	3.15e-12	6.84e-12	2.94e-05

*i* is an index for three partitions (groupings) of the data based on amino acid physiochemical properties. 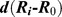 is the observed distance between a *i* ^th^ group-specific rate matrix (*R_i_*) and the rate matrix for the complete data (*R*_0_). 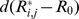
is the distance between a the *j* ^th^ random partition (denoted by *) of the complete data and the and the rate matrix for the complete data (*R*_0_). *j* is an index of 50 different random partitions of the data. The *t*-statistic is for a one-sample *t*-test. *p*_Bonf_ is the Bonferroni adjusted *p*-value.

**Figure 5:**
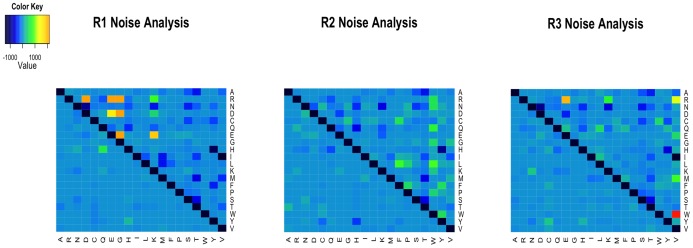
Heatmaps showing the difference between the group-specific rate matrices (*R*_1_, *R*_2_, *R*_3_) and the rate matrix estimated from the complete dataset (*R*_0_). The upper right triangle gives the difference between a partition derived from K-means clustering of the mammal data and mtMamR0. The lower left triangle gives the difference between a random grouping of mammalian sites and mtMamR0. Random groupings were constrained to the same size as the groups obtained by using K-means clustering. Panel (A) is for group 1 (1750 sites). Panel (B) is for group 2 (1025 sites). Panel (C) is for group 3 (805 sites).

To visualize the pattern that arises from a random partition we constructed heat maps of the difference between matrices on an element-by-element basis. Each element in a given heat map (Figure 5) represents the difference between an amino acid exchangeability estimated for a partition and the same exchangeability in the reference matrix (mtMamR0 or mtFishR0). The upper triangle of each matrix gives the difference between the reference matrix and the matrix for a physiochemically-defined group, and the lower triangle gives the difference between the same reference matrix and the matrix for the random grouping of sites of equal size. These heat maps clearly indicate that random partitioning of sites into groups leads to *R* matrices that are very similar to the reference matrix (*R*0), whereas the unsupervised grouping according to physiochemical properties leads to matrices that have unique differences from *R*0 (Figure 5).

### Cross validation

As expected, the group-specific *R* matrices provide a substantial improvement in explanatory power. However, some improvement in likelihood is expected even if there were no biological basis to the partitioning, as we fit a very parameter rich model (189 exchangeability parameters) to each group of sites. Hence, we employ cross validation to assess if the gains in likelihood associated with each of the partition-specific matrices (table 3) are also obtained if we apply the models to independent data. The procedure is a 50% cross validation of the likelihood score. The three clusters are randomly split into two equally sized subsets that are subsequently treated as training (T) and validation (V) sets. Typically, with *k* = 3 groups, the data can be paired according to the three groups (*i.e*., [T_1_,V_1_], [T_2_,V_2_], and [T_3_,V_3_]), and the R matrices are estimated from T*_i_* via maximum likelihood and applied to V*_i_*. In this case we use cross validation to confirm the overall pattern we observe in table 3, so we apply all three training matrices (R_T1_, R_T2_, and R_T3_), as well as the independently estimated *R* for the complete data (mtMamR0 or mtFishR0) to each validation dataset (V*_i_*). This is a computationally costly procedure due to the estimation of the exchangeability parameters in each T*_i_*. For this reason we carry out 10 replications of the cross validation procedure.

Results for both the mammal and fish datasets are very similar. For a given replication, the fit of the various matrices to the validation sets has the same pattern as in table 3; *i.e*., the partition-specific matrix out performs all the other matrices, with the joint *R* matrix (mtMamR0 or mtFishR0) providing the second best fit to each group of sites. Table 5 provides an example from one replication of the cross validation procedure, and table 6 provides the mean difference and standard deviation in likelihood scores over all 10 replicates. Note that this is not the standard use of cross validation, as the placement of sites into groups was derived from an analysis of the complete dataset prior to training. However, in this setting we are interested in the complete data estimate of the rate matrices (*R*_1_, *R*_2_, and *R*_3_) because we are supplying these as the best estimates of empirical matrices intended for use with other data sets (such as mtMam, mtArt, mtZoa and others are currently being used by the wider community). These results indicate that future analyses of either fish or mammal will likely benefit from the use of these matrices, as the joint R matrices do not represent the variation in physiochemical constraints among the sites of mitochondrial proteins.

**Table 5:** Results from one replicate of the 50% cross-validation of the likelihood score for alternative rate matrices (*R*_i_).

Mammal dataset			
	V_1_	V_2_	V_3_
*R*_0_	−58806.87	−39349.27	−15983.35
*R*_1_	**−52753.04**	−55574.13	−21695.61
*R*_2_	−68451.01	**−33077.13**	−18441.30
*R*_3_	−70395.13	−50302.24	**−12783.96**

Fish dataset			
	V_1_	V_2_	V_3_
*R*_0_	−29642.99	−20263.32	−9210.31
*R*_1_	**−26511.69**	−25587.05	−10915.49
*R*_2_	−33793.48	**−16537.54**	−11009.97
*R*_3_	−38222.13	−23863.04	**−7367.60**

Each group of sites was randomly divided into a training (*T_i_*) and a validation subset (*V*_i_). The likelihood score for *V*_i_ were obtained by using MLEs estimated from *T_i_*. The procedure was carried out for 10 replicates, results are provided above from one replicate as an example. The best likelihood scores are shown in bold.

**Table 6:** Mean and standard deviation (in parentheses) of difference in log-likelihood between group-specific and alternative rate matrices (*R_Ti_*) as applied to the validation datasets (*V*_i_) of the cross validation procedure.

Mammal dataset					
				*R_Ti_*	
	Group-				
*V*_i_ Data	Specific *R*	*R_0_*	*R_T1_*	*R_T2_*	*R_T3_*
*V*_1_	*R_T_*_1_	6087 (298)	-	15000 (730)	17415 (754)
*V*_2_	*R_T_*_2_	6238 (290)	22985 (1279)	-	17040 (965)
*V*_3_	*R_T_*_3_	3561 (235)	9522 (688)	6374 (429)	-

Fish dataset					
				*R_Ti_*	
	Group-				
*V*_i_ Data	Specific *R*	*R_0_*	*R_T1_*	*R_T2_*	*R_T3_*
*V*_1_	*R_T_*_1_	3074 (119)	-	7528 (695)	14533 (3607)
*V*_2_	*R_T_*_2_	3436 (287)	10477 (1272)	-	7437 (612)
*V*_3_	*R_T_*_3_	1900 (101)	4411 (621)	3957 (433)	-

*R*_0_ is the rate matrix estimate from a complete set of real amino acid sequences. *R_Ti_* is a rate matrix estimated from a group-specific training dataset. The cross validation procedure was based on 10 replicates of 50% cross validation.

**Figure 6:**
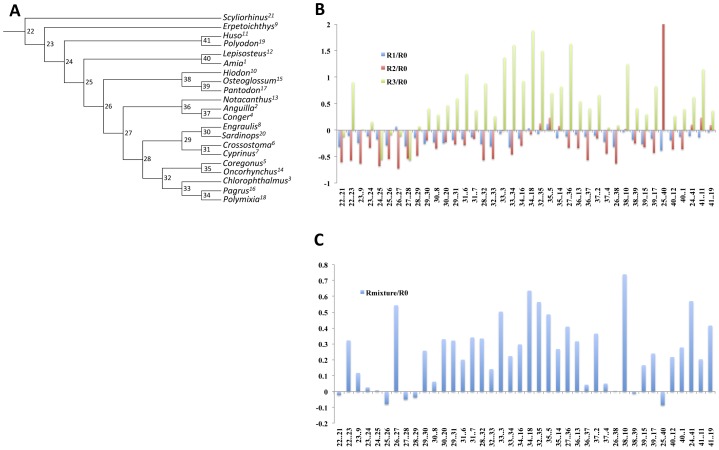
Comparison of branch lengths estimated under group-specific matrices and an overall matrix. Reduced datasets were used to investigate the impact of model-choice on branch lengths. **A**. A phylogenetic tree for 21 fish taxa. **B**. A plot showing differences between branch lengths estimated under partition-specific matrices and the mtFishR0 matrix. **C**. A plot showing the differences between the corrected branch lengths under a mixture of the partition specific matrices and mtFishR0 for the whole data. Differences between branch lengths (Bl) are measured as (Bl_*R_i_/*Bl_*R_0_*) – 1, where Bl_*R_i_* denotes branch lengths obtained using a partition specific matrix, and Bl_*R_0_* denotes branch lengths obtained using the reference matrix (mtFishR0). This measure centers the difference between branch lengths on 0, with values above 0 indicating branches that were larger under the partition-specific matrix and values below 0 indicating branches that were shorter under the partition-specific matrix. A value of zero indicates no difference between branch lengths.

Examination of the branch length differences between the estimated matrices and the mtMam for mammal data or mtFishR0 for the fish data showed variation throughout the tree. As applied to the appropriate sites, branch lengths estimated under R1 and R2 were generally shorter than when the overall matrix (mtMam or mtFishR0) was applied to the same group of sites. R3 gave different results, with some branches being longer when R3 matrix was used. This was more prominent in the fish than in the mammal dataset with many more branches affected. These findings are difficult to visualize given the large number of branches in the full datasets. To aid visualization of this result, we assembled two reduced datasets (22 taxa for the mammals, figure S2A, and 21 taxa for the fish, figure 6A). Results for the reduced datasets are similar to the full data and are shown in figure 6B (fish dataset) and figureS2B (mammal dataset). Interestingly the branches that seemed to be affected the most were internal branches (figure 6B and figureS2B).

Results presented in figure 6B and figure S2B isolate the impact of the exchangeabilities, as the reference set of branch lengths (from *R*0) were separately estimated by using the empirical amino frequencies for each site-group (*i.e.*, the frequencies were not miss-specified in *R*0). However, *R*0 would normally be applied using frequencies averaged over the complete data. To investigate complete data estimates of branch lengths, we mixed the MLEs of branch lengths under *R*1, *R*2, and *R*3 according to the frequencies of the three site-groups in the data and compared them to the complete-data estimates of branch lengths under *R*0 + average frequencies. As expected, there was a greater tendency for branch lengths to be smaller under a very simple model (R0 + average frequencies), as compared to the mixed-estimates (figure 6C and figure S2C). Although less prominent than in previous analyses, the impact on branch lengths was variable throughout the tree.

## Conclusions

We employed our new method to identify three large groups of sites evolving under unique physiochemical signatures. Interestingly, results were similar for both of the datasets examined in this study. Gap-statistics suggested *k* = 3 in both cases, and although the physiochemical signatures differed between groups of sites within a dataset, the group-specific signatures were similar between mammals and fish. We clustered sites into groups according to similarities in the physiochemical properties because we assumed that sites within a group were subject to unique evolutionary constraints. This notion was supported by the ML estimates of amino acid exchangeabilities for the different groups of sites. The noise-analysis indicated that the observed differences in amino acid exchangeabilities are significant. Although joint matrices such as mtMam and mtFishR0 perform reasonably well because they aggregate information over all sites, we found that they did not adequately represent the natural variability in the evolutionary processes among sites of mitochondrial proteins. Indeed, cross-validation indicated that models for mitochondrial protein data are significantly improved by the addition of site-class specific exchangeability matrices.

The most immediate application of this work is in the field of mitochondrial phylogenomics. Phylogenetic analysis of genomic data is commonly carried out under a “partition model”, although usually at the DNA sequence level (*e.g*., [31]–[33]). In a partition model, the data are divided *a priori* into subsets, typically whole genes, and independent model parameters are employed for the different partitions. Users of partition models are assuming (1) that there are significant differences among groups of sites in the evolutionary process, and (2) that they know which sites belong to which group with little or no error (but see [33] for an alternative approach). Partition models are attractive because they are computationally less costly than mixture models [32]. Unlike other empirical models for amino acid data, our set of site-class specific matrices will permit amino acid level analysis of mitochondrial data without having to assume an identical amino acid exchangeabilities for all sites. To facilitate this, we provide on-line (table S1), a map of our site-class specific matrices to the sites in our MSAs. Thus, if a new sequence can be aligned to one of our aligned sequences, the appropriate exchangeability matrix can be identified for each site in that new sequence. In addition to specification of a site-class specific matrix, we suggest that partition models should also include group-specific empirical amino acid frequencies and a branch length scale parameter. Programs such as RAxML [34] can be adapted to this purpose and then used to search tree space.

An alternative, and more computationally costly, approach to phylogenomics is to use a mixture model. Here the user is still assuming that there are significant differences among groups of sites in the evolutionary process, but they are no longer willing to assume they know which sites belong to a given group within the model. Recent models for amino acid sequences mix at both the level of the matrix and the level of the evolutionary rate [17], [18], but they have not yet been widely adopted. In those models, the likelihood of the data is computed as a weighted average over a set of matrices (or amino acid frequency profiles) and over the standard rate categories of a gamma model [9], [10]. Using the analytical framework developed by [17] or [18], mixture models could be constructed for mitochondrial data by employing our set of site-class specific matrices in place of the matrices used in their models. Because these models average over matrices (and rate categories), a user will not be required to specify which site belongs to a given matrix within the model as is the case with the partition models.

There is a wide range of other mitogenomic-based research activities that could benefit from improved modeling. Phylogeny-based approaches for estimation of divergence dates, or the intensity of functional divergence, are particularly noteworthy. Mitochondrial data are often used to infer divergence dates (*e.g*., [4]–[6]). Partition models, again usually applied to DNA level analyses, are becoming widely recognized as important (*e.g*., [35], [36]). Because we found that inadequate modeling of mitogenomic data can negatively impact ML estimates of branch lengths, we expect that estimation of deeper divergence times from such data could likewise be negatively impacted. Mitogenomic data are also the focus of analyses for functional divergence (*e.g*., [37], [38]). Recent work on model-based methods to test for functional divergence have begun to employ amino acid exchangeability matrices as a means of improving the involved statistical tests [39], [40]. However, those methods employ a single matrix to model the effect of physiochemcial properties on the amino acid replacement rate. A possible negative outcome of this modeling strategy is that un-modeled variation among sites could be incorrectly “soaked up” by some of the other parameters in those models, and this could impact tests that depend on reliable estimates of parameter values [41]. Although the site-class specific matrices estimated in this study are suitable only for mitochondrial data, the underlying modeling issues are relevant to the analysis of other types of data.

The impact of site-class specific matrices on phylogenomic inference, divergence date estimation, and studies of functional divergence are important directions for further research, but are beyond the scope of this study. Within the context of those activities, it will be interesting to explore the effect of clustering according to alternative measures of physiochemical properties. Furthermore, we expect that site-class specific exchangeability matrices will differ among the more divergent lineages of metazoans (*e.g*., cnidarians, arthropods, lophotrochozoans), as has been observed among joint matrices (*e.g*., [20]–[22]). Beyond the more practical benefits to these research activities, clustering of sites and estimating exchangeabilities can be used to directly investigate questions of molecular evolution. For example, the approach could shed some light on the relative importance of the genetic code versus physiochemical constraints in explaining the differences observed between the more divergent lineages of metazoans. Because the adequacy of an evolutionary model is central to so many different research activities, we predict that our general approach to grouping sites for the purpose of estimating exchangeabilities could have value beyond mitogenomic datasets.

## Methods

### Data sets

All analyses are carried out on two datasets. The mammalian dataset is comprised of the amino acid sequences from 12 mitochondrially-encoded genes sampled from 143 lineages. The fish dataset is comprised of 11 mitochondrially-encoded genes sampled from 75 lineages of Actinopterygians (ray-finned fishes). A list of all the organisms, and the accession numbers for their complete mitochondrial genomes, is provided in table S3. All the protein-coding genes were parsed from the genome sequence and the sequences of the 12 genes encoded on the heavy strand were translated and aligned by using the program t-coffee [42]. Alignments (table S1) were visually inspected and regions having questionable positional homology were either adjusted manually or removed from the MSA. Those few amino acids that are encoded by overlapping reading frames also were removed from the MSA. Additionally, ATPase 8 was removed from the fish dataset because most of its sites overlap with the sites of adjacent genes. The resulting MSA for the mammal and fish datasets were comprised of 3580 and 3370 amino acid sites respectively. Additional alignment details are provided supplemental methods S1.

### K-means clustering and gap statistics

K-means [43] is a relatively simple procedure for unsupervised learning, having the advantage of a very fast operation time. We employed the Hartigan and Wong algorithm [44], as implemented in the program R [45]. We employ this algorithm for the purpose of grouping sites in a MSA according to similarities in their physiochemical properties. The algorithm is applied to vectors containing nine different physiochemical property values that correspond to the mean values of the amino acids at a specific site in a MSA. As K-means is a hill-climbing algorithm, we use 1000 different random initial assignments and the results that minimized the Euclidean distance of each member of a group from the physiochemical centroid of that group are taken as the best result.

To determine the best number of groups for the data in hand we used a gap statistic. The gap is a measurement of the difference between the error within a cluster (denoted as *W_k_*) and its expected value under the reference, or null, distribution. The first step is to create a uniform distribution on the results of a singular value decomposition of the matrix. The reference distribution is then obtained by transforming this uniform back to the original dimensions [28]. Monte Carlo samples are drawn from the reference distribution such that the gap can be measured as:
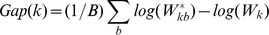



where *k* = 1, 2, 3 … *K* groups, or clusters, and *b* = 1, 2, 3, … *B* reference features derived from the reference distribution from which log (*W_k_*) is estimated. Thus 
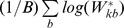
 is an estimate of the expected value of log (*W_k_*). The value of *k* is then chosen to be the smallest *k* where *Gap(k) ^3^ Gap(k+1)−S_k+1_* is satisfied. Where *S_k_* is the standard deviation of the log of the distance vectors of the reference data under *k* clusters, and *Gap(k)* is based on *B* = 100. This, selection of *k* follows the “1-standard-error” rule of [28].

### ML estimation of amino acid exchangeabilities

The substitution rate matrix for amino acids (*Q*) contain the instantaneous rates of change from amino acid *i* to amino acid *j* (*q_ij_*), where *i* and *j* index the 20 different amino acids. The off-diagonal elements of matrix *Q* is described by the product of a symmetric matrix of amino acid exchange-rate parameters 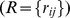 and a diagonal matrix of equilibrium frequencies 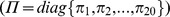; thus 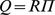. We follow Whelan and Goldman [7] by referring to the 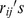 as exchangeability parameters for pairs of amino acids (*i, j*).

All 189 parameters from the matrix, *R*, were determined under a maximum likelihood (ML) framework using the computer program codeml from the PAML package [9]. ML estimation of *R* was carried out using a fixed tree topology. The topology was estimated from the same data (but excluding third codon positions) by using a neighbor-joining analysis of pairwise distances computed under the HKY85+discreteGamma model. In the case of the fish dataset, ML estimation was also carried out under a topology derived from morphological characters [30]. All tree topologies are provided on-line as supplementary information in the nexus format (TreefileS1 and TreefileS2). Two different techniques were used to estimate the exchangeability parameters. The first involved jointly estimating the 189 parameters at the same time as the estimation of the branches lengths for the tree (284 branch length parameters for the mammal tree and 184 for the fish tree). This represents a large computational burden. The second method cycled iteratively between two phases of optimization. In the first phase, branch lengths were estimated under a fixed set of exchangeability parameter values, and in the second phase the exchangeability parameter values were estimated under a fixed set of branch lengths. The second method cycled between the two phases until convergence. Both methods require the optimization to start from a set of initial parameter values, and we found that the optimization could be sensitive to the initial values of the exchangeability parameters in some cases. Hence every matrix was estimated by using both methods and multiple sets of initials for the exchangeability values (the empirical matrices mtMam, mtRev24, Grantham, JTT, and WAG were used as different sets of initial values). The results from each run were examined and the best set of exchangeabilities was determined according to the likelihood score. Analyses were performed using a 20 node dual core Opteron 270, 2.2GHz, 4GB system running freebsd. The time required to obtain a rate matrix varied based on size of partition and method used. Smaller partitions required half a day to complete using method 1, to a week using the second method. The large partition took 2 weeks using the first method and a month with the second method.

### Noise analysis

K-means clustering with gap-statistics yields *k* subsets of an original MSA, and ML is used to infer an R matrix specific to each subset. Noise analysis is carried out here to assess if differences between *R* matrices estimated for the *k* subsets of the data differ from a reference *R* matrix in excess of what would have been observed if the sites had been randomly partitioning among the *k* subsets. If the *k* subsets possess *N*_1_, *N*_2_, … *N_k_* sites separately, then *S* replicates of random subsets having sizes *N*_1_, *N*_2_, … *N_k_* are generated for the noise analysis. Due to heavy computational cost of analyzing the random subsets of the data we employ *S* = 50 replicates, leading to 50×*k* subsets for which we must estimate an *R* matrix via ML as described above. Such matrices are denoted as 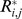, with *i* indexing 1, 2, 3, … *k* subsets of the data, and *j* = 1, 2, 3, …50 replicates. Each 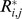 is compared to a reference *R* matrix denoted as *R*_0_. Hence, we measure a distance denoted 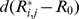 for each of 50×*k* random subsets of the MSA and this serves as a baseline to which we compare 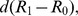 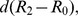 …, 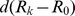.

We employ the following distance statistic to measure the difference between a particular subset-specific matrix (either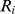 or 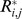) and a reference matrix (*R*_0_):
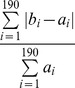



where *a_i_* is the *i* ^th^ entry in the reference matrix and *b_i_* is the corresponding entry in the subset-specific matrix. For a given subset of the MSA and *S* = 50 replicates, we compute 51 values of this distance; one for 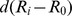
and 50 for 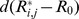. A one-sample *t*-test is used to determine if the mean of the 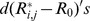
is less than 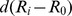.

## Supporting Information

FigureS1 **Similarity between mtManR0 and mtMam matrices of amino acid exchangeabilities.** The mtMam matrix contains the amino acid exchangeabilities for mammalian mitochondrial sequences estimated by [21]. The mtMamR0 matrix contains the amino acid exchangeabilities for mammalian mitochondrial sequences estimated in this study. Both matrices aggregate evolutionary process information over all sites. The estimated exchangeabilities are very similar between mtMam and mtMamR0.
(PDF)

Figure S2 **Comparison of branch lengths estimated under group-specific matrices and an overall matrix.** Reduced datasets were used to investigate the impact of model-choice on branch lengths. **A**. A phylogenetic tree for 22 mammals. **B**. A plot showing differences between branch lengths estimated under partition-specific matrices and the mtMam matrix. **C**. A plot showing the differences between the corrected branch lengths under a mixture of the partition specific matrices and mtMam for the whole data. Differences between branch lengths (Bl) are measured as (Bl_*R_i_/*Bl_*R_0_*) – 1, where Bl_*R_i_* denotes branch lengths obtained using a partition specific matrix, and Bl_*R_0_* denotes branch lengths obtained using the reference matrix (mtMam). This measure centers the difference between branch lengths on 0, with values above 0 indicating branches that were larger under the partition-specific matrix and values below 0 indicating branches that were shorter under the partition-specific matrix. A value of zero indicates no difference between branch lengths.
(PDF)

TableS1 **The multiple sequence alignments (MSAs) for the mammal and fish datasets.** The MSAs for the mammal and fish datasets are 3580 and 3370 amino acid sites respectively. These MSAs are provided as separate worksheets within a single excel file. The second line of each MSA is a column-specific indicator variable that gives the assignment of each site in the MSA to a group-specific R matrix; 1 = large group (1750 in mammals or 1607 in fish); 2 = medium (1025 in mammals or 999 in fish); 3 = small (805 in mammals or 764 in fish).
(XLSX)

Table S2 **Likelihood of full dataset and three partitions based on a rate matrix estimated from the complete data (R0) and three partition-specific rate matrices (R1, R2 and R3).** The original branch lengths for the analysis are fixed to those obtained from the total data using R0 and then a scaling factor is applied
(DOCX)

TableS3 **List of the GenBank accession numbers for the complete mitochondrial genomes of all organisms used in this study.** The mammalian dataset is comprised of amino acids from the mitochondrial genomes of 143 linages of mammals. The fish dataset is comprised of amino acids from the mitochondrial genomes of 75 linages of Actinopterygians (ray-finned fishes).
(XLSX)

PmatrixS1 **Physiochemical property matrices for the mammal dataset.** The matrix is a numerical representation of the mean physiochemical properties of the amino acids at each site in the mammal MSA. The matrix is *n*×*m*, because *m* different mean physiochemical properties are computed for *n* different sites in the original MSA. This matrix is for the mammal dataset and is 3580×9.
(TXT)

PmatrixS2 **Physiochemical property matrices for the fish dataset.** The matrix is a numerical representation of the mean physiochemical properties of the amino acids at each site in the fish MSA. The matrix is *n*×*m*, because *m* different mean physiochemical properties are computed for *n* different sites in the original MSA. This matrix is for the fish dataset and is 3370×9.
(TXT)

RmatricesS1 **Matrices of amino acid exchangeabilities specific for groups of sites in mammalian mitochondrial proteins having different physiochemical constraints.** Three matrices were estimated for three groups of sites that were identified by K-means clustering according to mean physiochemical properties (PmatrixS1). The R matrix for the largest group (1750 sites) is called mtMamR1. The R matrix for the medium sized group (1025 sites) is called mtMamR2. The R matrix for the smallest group (805 sites) is called mtMamR3.
(RTF)

RmatricesS2 **Matrices of amino acid exchangeabilities specific for fish mitochondrial proteins.** Three matrices were estimated for three groups of sites that were identified by K-means clustering according to mean physiochemical properties (PmatrixS2). A fourth matrix was jointly estimated from all sites. Due to disagreements in the phylogenetic relationships of fishes, these four matrices were estimated under two alternative tree toplogies, one based on molecular data (designated by “mol”) and one based on morphological data (designated by “morph”). Thus, this file contains eight matrices of amino acid exchangeabilities. The R matrix for the largest group (1607 sites) is called mtFishR1. The R matrix for the medium sized group (999 sites) is called mtFishR2. The R matrix for the smallest group (764 sites) is called mtFishR3. The fourth matrix contains exchangeabilities jointly estimated for all sites in the dataset (3370) and is called mtFishR0. The labels “mol” or “morph” indicate the tree topology used to estimate the matrix.
(RTF)

MethodsS1 **Sequence alignment methods.** This supplemental methods section provides a detailed description of the protocol for sequence alignment. In addition, the method of post-alignment filtering of sites is described and a list of sites excluded from subsequent analysis is provided.
(PDF)

TreefilesS1 **Mammal tree topology in nexus file format.** Phylogenetic tree topology estimated from the full alignment of 3580 amino acid sites from 143 linages of mammals (supplementary TableS2).
(TXT)

TreefilesS2 **Fish tree topologies in nexus file format.** This file contains two nexus-formatted tree topologies. The first is the phylogenetic tree topology estimated from the full alignment of 3370 amino acid sites from 75 lineages of fish (supplementary TableS2). The second is an alternative topology for these same lineages derived from morphological data [30]. The morphological and molecular topologies differ in two places. One is the relationship between gars, sturgeons, and amiids. The other is the position of anguilliforms and osteoglossiforms.
(TXT)
