# Supplementary figures and images for: Improving Evolutionary Models for Mitochondrial Protein Data with Site-Class Specific Amino Acid Exchangeability Matrices
Source: PLoS One. 2013 Jan 31;8(1):e55816. doi: 10.1371/journal.pone.0055816 (PMC3561347; doi:10.1371/journal.pone.0055816)

FigureS1

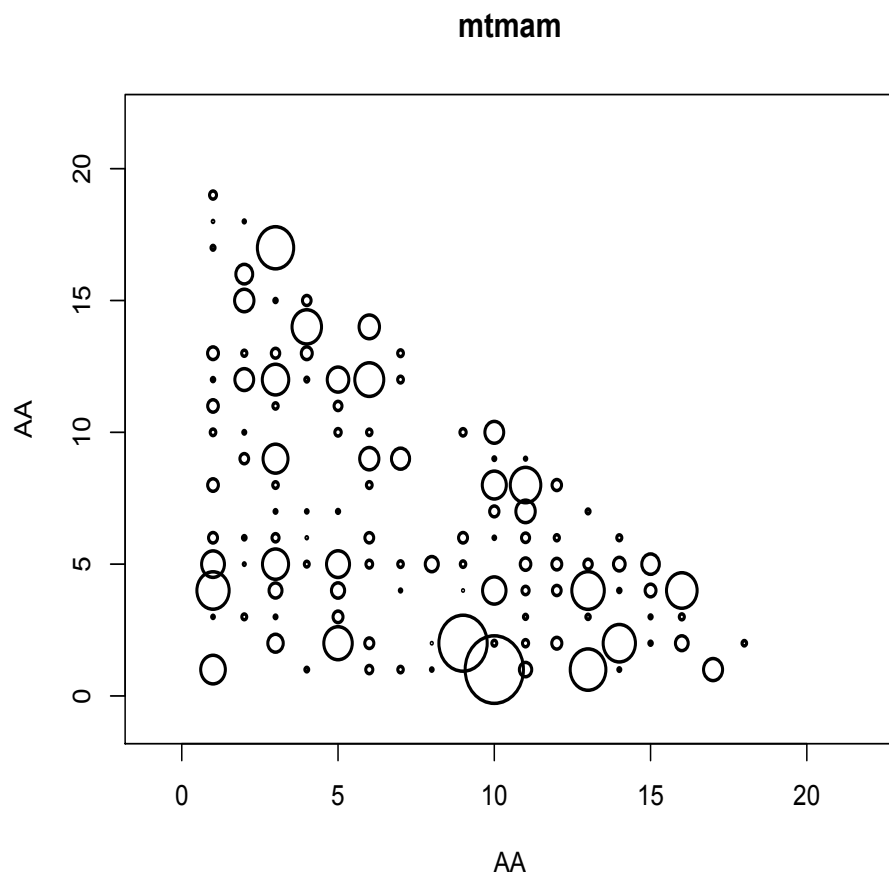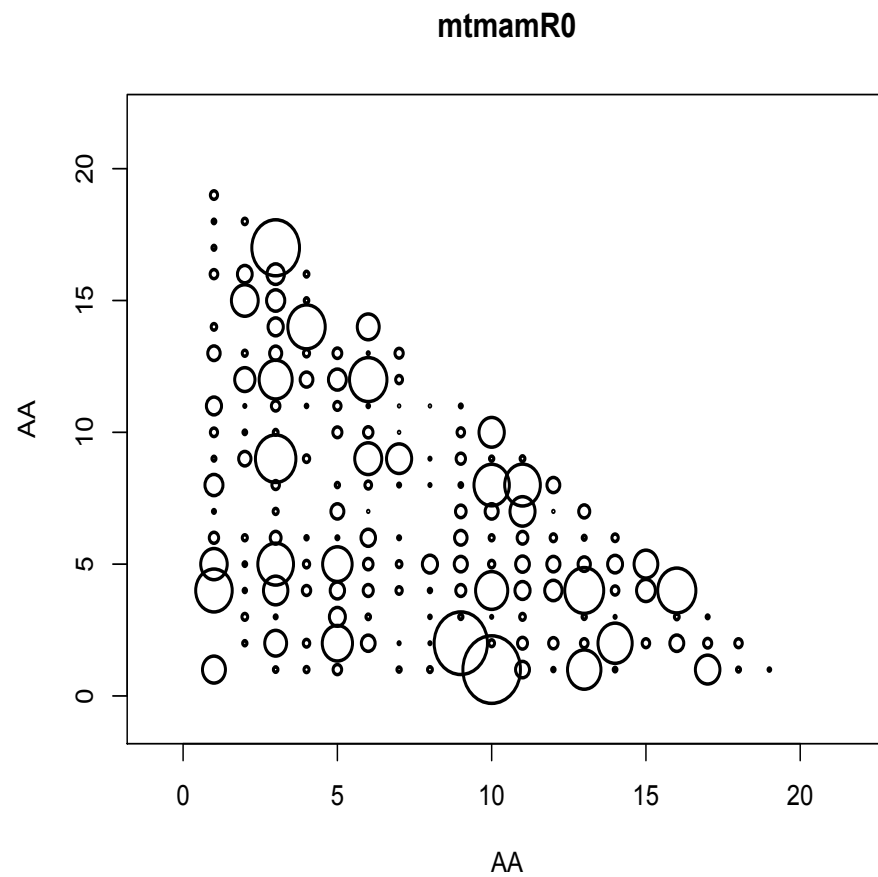

Supplement: FigureS1 — Similarity between mtManR0 and mtMam matrices of amino acid exchangeabilities. The mtMam matrix contains the amino acid exchangeabilities for mammalian mitochondrial sequences estimated by [21]. The mtMamR0 matrix contains the amino acid exchangeabilities for mammalian mitochondrial sequences estimated in this study. Both matrices aggregate evolutionary process information over all sites. The estimated exchangeabilities are very similar between mtMam and mtMamR0. (PDF) [file pone.0055816.s001.pdf]

**A**

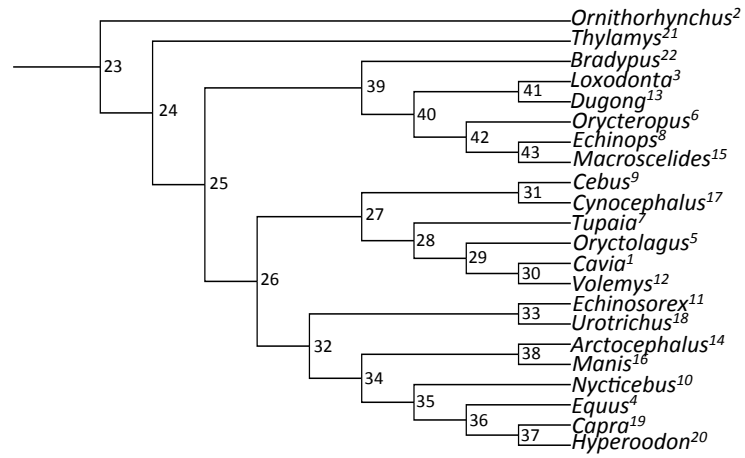

**B**

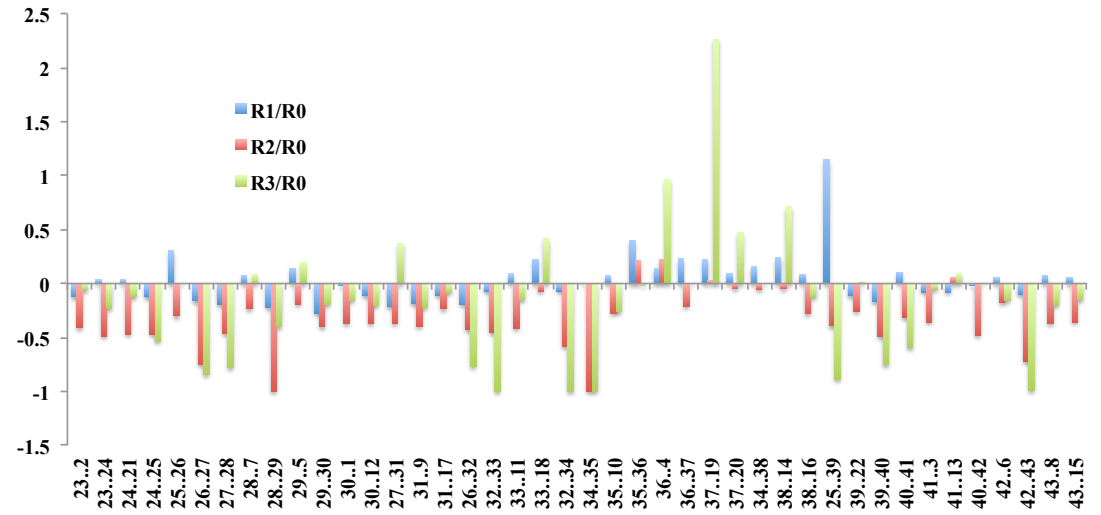

**C**

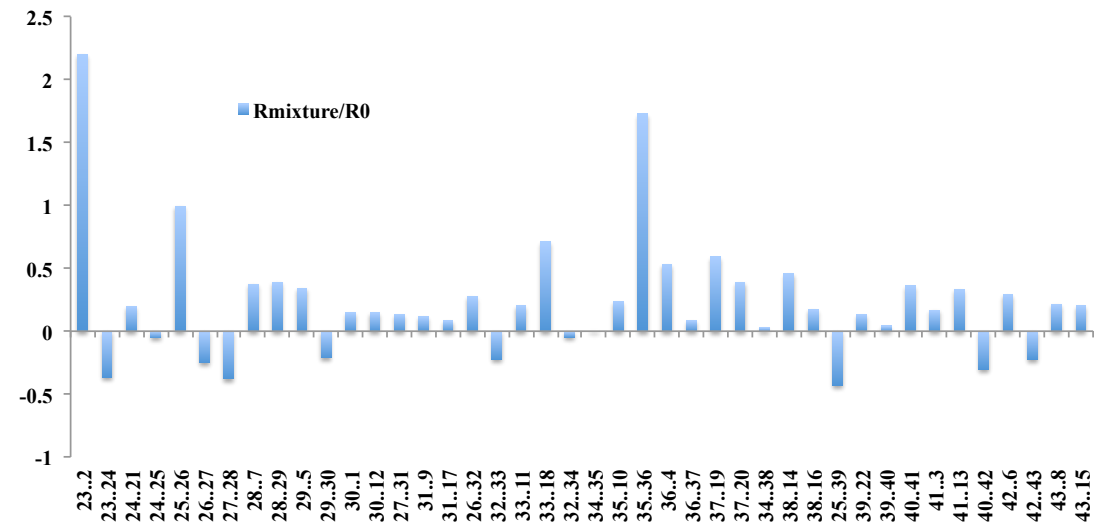

Supplement: Figure S2 — Comparison of branch lengths estimated under group-specific matrices and an overall matrix. Reduced datasets were used to investigate the impact of model-choice on branch lengths. A. A phylogenetic tree for 22 mammals. B. A plot showing differences between branch lengths estimated under partition-specific matrices and the mtMam matrix. C. A plot showing the differences between the corrected branch lengths under a mixture of the partition specific matrices and mtMam for the whole data. Differences between branch lengths (Bl) are measured as (Bl_Ri/Bl_R0) – 1, where Bl_Ri denotes branch lengths obtained using a partition specific matrix, and Bl_R0 denotes branch lengths obtained using the reference matrix (mtMam). This measure centers the difference between branch lengths on 0, with values above 0 indicating branches that were larger under the partition-specific matrix and values below 0 indicating branches that were shorter under the partition-specific matrix. A value of zero indicates no difference between branch lengths. (PDF) [file pone.0055816.s002.pdf]
