# Supplementary material for: Improving Evolutionary Models for Mitochondrial Protein Data with Site-Class Specific Amino Acid Exchangeability Matrices
Source: PLoS One. 2013 Jan 31;8(1):e55816. doi: 10.1371/journal.pone.0055816 (PMC3561347; doi:10.1371/journal.pone.0055816)
Supplement: Table S2 — Likelihood of full dataset and three partitions based on a rate matrix estimated from the complete data (R0) and three partition-specific rate matrices (R1, R2 and R3). The original branch lengths for the analysis are fixed to those obtained from the total data using R0 and then a scaling factor is applied (DOCX) [file pone.0055816.s004.docx]

**Table S2.** Likelihood of full dataset and three partitions based on a rate matrix estimated from the complete data (R0) and three partition-specific rate matrices (R1, R2 and R3). The original branch lengths for the analysis are fixed to those obtained from the total data using R0 and then a scaling factor is applied.

|  |  | |  | |  | | | |  | | | |  | |
| --- | --- | --- | --- | --- | --- | --- | --- | --- | --- | --- | --- | --- | --- | --- |
| Mammal dataset (3580 sites) | | |  | |  | | | |  | | | |  | |
|  | |  | | **Subset of data**  **(no. of sites)** | | | | | | | | | | |
| *R* matrix | | **Full data** | | **Group 1**  **(1750)** | | | | **Group 2**  **(1025)** | | | | **Group 3**  **(805)** | | |
| mtMamR0 | | **-216607.19 (1)** | | -119368.26 (1.16) | | | | -76540.89 (1.19) | | | | -35565.02 (0.57) | | |
| mtMamR1 | | -334533.78 (1.35) | | **-109325.69 (1.15)** | | | | -110215.93 (1.38) | | | | -46862.44 (0.66) | | |
| mtMamR2 | | -256130.93 (1.03) | | -137511.59 (1.15) | | | | **-63852.61 (1.25)** | | | | -41026.73 (0.55) | | |
| mtMamR3 | | -274610.01 (1.16) | | -140809.19 (1.29) | | | | -96871.41 (1.33) | | | | **-29129.15 (0.54)** | | |
|  | | | |  | | |  | | |  | | | |  |
| Fish dataset (3370 sites) | | | |  | | |  | | |  | | | |  |
|  | |  | | **Subset of data**  **(no. of sites)** | | | | | | | | | | |
| *R* matrix | | **Full data** | | **Group 1**  **(1607)** | | **Group 2**  **(999)** | | | | | **Group 3**  **(764)** | | | |
| mtFishR0 | | **-110263.36 (1)** | | -58831.98 (1.18) | | -40009.17 (1.15) | | | | | -19424.74 (0.59) | | | |
| mtFishR1 | | -177312.61 (1.18) | | **-53491.07 (1.05)** | | -51985.39 (1.24) | | | | | -24852.55 (0.69) | | | |
| mtFishR2 | | -137348.43 (1.04) | | -67372.48 (1.15) | | **-32921.43 (1.30)** | | | | | -23324.11 (0.60) | | | |
| mtFishR3 | | -148512.04 (1.21) | | -74231.67 (1.42) | | -47930.25 (1.24) | | | | | **-15664.60 (0.65)** | | | |

The best likelihood score is shown in bold. Scaling factors are shown in parentheses.
