# Supplementary material for: Improving Evolutionary Models for Mitochondrial Protein Data with Site-Class Specific Amino Acid Exchangeability Matrices
Source: PLoS One. 2013 Jan 31;8(1):e55816. doi: 10.1371/journal.pone.0055816 (PMC3561347; doi:10.1371/journal.pone.0055816)
Supplement: RmatricesS1 — Matrices of amino acid exchangeabilities specific for groups of sites in mammalian mitochondrial proteins having different physiochemical constraints. Three matrices were estimated for three groups of sites that were identified by K-means clustering according to mean physiochemical properties (PmatrixS1). The R matrix for the largest group (1750 sites) is called mtMamR1. The R matrix for the medium sized group (1025 sites) is called mtMamR2. The R matrix for the smallest group (805 sites) is called mtMamR3. (RTF) [file pone.0055816.s008.rtf]

 RmatricesS1mtMamR1Arg	62.04																			
Asn	0.99	17.49																		
Asp	30.81	2161.62	32.18																	
Cys	0	1.1	1.05	0																
Gln	0	0	45.27	95.65	0															
Glu	16.83	2161.62	0	2161.62	2.55	14.97														
Gly	61.24	2161.62	1.61	2161.62	1.38	16.2	2161.62													
His	0	11.32	169.77	18.25	7.3	120.31	84.03	5.43												
Ile	2.14	46.21	1.14	0.98	0.01	0	3.2	31.88	0.32											
Leu	0.24	2.8	0	31.21	0.02	1.05	14.62	18.48	0.62	0.15										
Lys	71.71	896.53	156.48	385.88	1.27	56.97	2161.62	0	86.01	5.68	16.13									
Met	2.2	48	0.07	53.17	0	0	3.03	9.82	0	0.32	0.36	23.28								
Phe	0.16	0.61	0	0.35	0.07	0	54.95	3.17	3.19	0.03	0.24	2.87	0.01							
Pro	3.4	20.84	5.87	0	0	23.71	81.17	23.87	44.64	0.35	3.5	29.37	0.26	1.13						
Ser	14.31	0	39.15	17.79	6.01	0	84.37	10.92	16.4	0.15	0.82	176.4	0.01	2.04	62.31					
Thr	37.28	8.88	13.58	11.1	0.27	0.88	4.27	34.09	2.32	4.15	0.29	99.62	4.09	0.02	21.92	18.75				
Trp	0	0.1	0.65	0	0.16	0.74	0.24	0.32	1.12	0	0.01	0.43	0	0	0.37	0.28	0.01			
Tyr	0	0.05	0.21	0.47	0.31	0	3.91	0.06	42.6	0.01	0.01	0	0.01	0.64	0.55	0.24	0.01	0		
Val	5.41	6.37	0	0.49	0.05	0	0.4	10.78	0.06	2.16	0.07	0.62	0.59	0.01	0.39	0	1.98	0.01	0	
	Ala	Arg	Asn	Asp	Cys	Gln	Glu	Gly	His	Ile	Leu	Lys	Met	Phe	Pro	Ser	Thr	Trp	Tyr	
mtMamR2Arg	0.34																			
Asn	0.18	1.02																		
Asp	1.43	0.11	687.89																	
Cys	14.99	27.5	337.97	0.11																
Gln	0.11	2.54	11.74	25.47	0.11															
Glu	0.11	0.11	1.81	218.4	2.71	2.02														
Gly	72.99	12.69	189.54	20.42	321.35	1.83	33.01													
His	0.11	1.83	23.01	9	8.7	6.57	0.11	0.11												
Ile	4.74	0.11	124.38	21.42	113.47	0.41	1.23	54.82	0.11											
Leu	0.31	1.01	73.58	12.14	185.26	10.68	0.35	0.11	1.74	377.96										
Lys	0.11	0.24	79.53	19.09	0.11	2.09	2.69	7.2	0.11	3.19	1.85									
Met	6.68	0.11	35.38	0.11	30.98	1.41	0.97	0.11	0.11	335.13	500.91	18.22								
Phe	0.11	0.11	29.11	0.11	587.98	1.42	0.11	60.28	11.66	250.02	1107.56	0.11	20.63							
Pro	5.95	3.9	206.66	141.57	0.11	27.79	0.11	0.11	13.33	142.3	417.77	17.17	37.59	423.71						
Ser	21.32	0.11	137.99	50.76	242.11	0.84	0.11	173.44	0.73	74.4	134.13	1.94	64.68	188.71	202.09					
Thr	5.78	0.11	16.07	1.85	15.66	0.11	0.11	7.11	0.11	89.83	3.95	0.33	77.82	3.6	38.17	35.96				
Trp	0.11	514.74	500.05	80.73	0.11	749.39	0.11	423.95	83.08	75.42	913.37	312.94	0.11	0.11	398.72	0.11	0.11			
Tyr	0.11	6.87	344.64	80.22	561.86	27.77	0.11	0.11	217.35	51.35	156.02	2.84	0.11	981.57	162.72	59.91	0.76	488.71		
Val	137.92	0.64	10.63	53.27	0.11	0.11	1.35	258.36	0.11	1122.71	178.78	0.11	626.48	25.7	37.07	85.13	99.69	0.11	0.11	
	Ala	Arg	Asn	Asp	Cys	Gln	Glu	Gly	His	Ile	Leu	Lys	Met	Phe	Pro	Ser	Thr	Trp	Tyr	
mtMamR3Arg	42.28																			
Asn	0.01	47.1																		
Asp	0	0	0.41																	
Cys	0	0	0	0																
Gln	0	168.06	0	0	0															
Glu	2.94	2029.72	52.63	82.88	32.11	453.4														
Gly	2.44	60.48	0.01	0.02	0.33	0.04	0.82													
His	0	272.93	7.11	0.47	32.78	285.53	113.5	0												
Ile	53.2	0	13.76	0	32.05	53.35	91.51	0	44.44											
Leu	104.45	0	0	0.19	12.05	469.13	49.24	0	117.24	523.92										
Lys	0	792.3	15	0	0	113.59	226.48	0	56.29	51.52	32.54									
Met	76.62	0	0	0	151.34	96.67	0	0	0	441.16	577.58	29.02								
Phe	16.83	0	0	0	0	226.03	0	0	0	58.95	196.51	26.19	218.26							
Pro	0.46	2.13	0	0	0	7.77	0	0	0	0	46.54	0.35	0	0.26						
Ser	8.34	4.91	0.4	0	4.8	2.06	2.06	0.07	0.33	0.5	15	1.66	59.88	1.83	0.05					
Thr	58.01	18.87	0.29	0	0	21.85	36.68	0	0	139.77	91.73	10.01	328.46	0	0.18	1.61				
Trp	62.14	0	0	0	530.7	0	0	73.38	0	0	0	0	36.34	119.05	0	50.8	0			
Tyr	0	67.82	17.43	0.02	43.12	284.81	18.92	0.03	522.29	40.82	4.53	54.05	102.64	564.36	0	0.27	2.4	49.51		
Val	3.36	1287.73	110.69	7.64	134.48	1.17	450.1	0.53	133.94	2.77	228.13	59.77	1331.94	46.75	0	21.68	116.47	2765.47	129.68	
	Ala	Arg	Asn	Asp	Cys	Gln	Glu	Gly	His	Ile	Leu	Lys	Met	Phe	Pro	Ser	Thr	Trp	Tyr	
