# Supplementary material for: Improving Evolutionary Models for Mitochondrial Protein Data with Site-Class Specific Amino Acid Exchangeability Matrices
Source: PLoS One. 2013 Jan 31;8(1):e55816. doi: 10.1371/journal.pone.0055816 (PMC3561347; doi:10.1371/journal.pone.0055816)
Supplement: RmatricesS2 — Matrices of amino acid exchangeabilities specific for fish mitochondrial proteins. Three matrices were estimated for three groups of sites that were identified by K-means clustering according to mean physiochemical properties (PmatrixS2). A fourth matrix was jointly estimated from all sites. Due to disagreements in the phylogenetic relationships of fishes, these four matrices were estimated under two alternative tree toplogies, one based on molecular data (designated by “mol”) and one based on morphological data (designated by “morph”). Thus, this file contains eight matrices of amino acid exchangeabilities. The R matrix for the largest group (1607 sites) is called mtFishR1. The R matrix for the medium sized group (999 sites) is called mtFishR2. The R matrix for the smallest group (764 sites) is called mtFishR3. The fourth matrix contains exchangeabilities jointly estimated for all sites in the dataset (3370) and is called mtFishR0. The labels “mol” or “morph” indicate the tree topology used to estimate the matrix. (RTF) [file pone.0055816.s009.rtf]

RmatricesS2mtFishR1molArg	83.8																			
Asn	12.34	0																		
Asp	30.42	0	0																	
Cys	0	0	0	0																
Gln	35.55	720.15	114.36	1648.2	0															
Glu	76.77	0	0	1648.2	3.48	1648.2														
Gly	204.34	1648.2	0	0	0.1	1046.27	0													
His	0.66	0	0	0	6.62	111.6	0	10.34												
Ile	0.72	23.24	0	30.55	0.02	0	54.04	42.6	0.84											
Leu	0.23	26.62	0.16	0.82	0	24.77	3.06	1.7	1.56	0.21										
Lys	0	0	123.45	1648.2	1.8	703.75	0	1648.2	64.38	20.2	20.03									
Met	1.52	0	0	95.77	0	37.53	6.07	6.65	0.08	0.2	0.33	16.5								
Phe	0	0	0	0	0.01	0	10.67	0	18.73	0.02	0.11	1.04	0.01							
Pro	26.57	563.09	15.41	0	2.74	1109.03	1648.2	697.08	46.86	1.44	12.78	0	1.39	42.87						
Ser	77.67	0	131.9	0	11.09	30.61	0	48.55	71.72	1.2	1.09	73.62	2.61	4.68	25.84					
Thr	46.33	0	27.61	15.61	0	78.78	56.66	108.88	0	3.72	0.48	67.68	3.34	0	9.59	68.39				
Trp	0	0.06	0	0	0.09	0.17	0	0.29	2.4	0	0.02	0.29	0.01	0.01	0.43	0.93	0			
Tyr	0	0.35	4.58	0.78	0.03	0	0.7	0	84.61	0	0	0.5	0	0.22	0	0.41	0	0.01		
Val	3.01	10.72	0.73	23.69	0	0	1.12	32.09	0.28	1.65	0.08	2.96	0.2	0	2.64	0.09	2.74	0.01	0	
	Ala	Arg	Asn	Asp	Cys	Gln	Glu	Gly	His	Ile	Leu	Lys	Met	Phe	Pro	Ser	Thr	Trp	Tyr	
mtFishR1morphArg	33.74																			
Asn	10.12	0																		
Asp	20	1804.86	0																	
Cys	0	0	0	0																
Gln	0	0	0	0	0															
Glu	246.32	1804.86	0	1804.86	1.5	4.25														
Gly	31.01	526.03	0	85.97	4.22	0	1804.86													
His	0.93	0	0	0	6.97	61.82	0	77.89												
Ile	0.96	32.56	0	34.93	0.03	0	43.48	3.87	0.75											
Leu	0.27	27.48	0.12	3.19	0	0.8	1.57	15.3	1.66	0.21										
Lys	56.59	1804.86	0	1804.86	0.69	179.17	0	1662.58	54.11	2	26.2									
Met	1.48	3.35	0	92.4	0	2.3	7.06	2.34	0.14	0.23	0.33	60.85								
Phe	0.02	0	0	0	0.01	0	0.6	59.8	19.22	0.03	0.13	0	0.02							
Pro	51.09	167.21	196.61	0	0.38	262.44	1804.86	633.83	79.22	7.34	21.1	364.47	11.38	2.27						
Ser	79.04	0	144.35	0	11.89	0	43.51	13.48	72.38	0.69	0.6	0	2.67	4.36	100.07					
Thr	49.53	0	16.29	31.56	0	5.43	92.02	9.5	0	4.4	0.39	8.14	3.64	0	123.11	62.21				
Trp	0	0.07	0	0.16	0.09	2.58	0.12	0.82	2.56	0	0.02	0	0.01	0.01	0	0.97	0			
Tyr	0	0.31	4.57	0.73	0.03	1.43	0	0	86.66	0	0	0	0	0.23	0.19	0.45	0	0.01		
Val	3.3	10.95	0.8	23.15	0	0.81	25.79	3.34	0.29	1.81	0.08	0	0.21	0	4.09	0.12	2.99	0.01	0	
	Ala	Arg	Asn	Asp	Cys	Gln	Glu	Gly	His	Ile	Leu	Lys	Met	Phe	Pro	Ser	Thr	Trp	Tyr	
mtFishR2molArg	0.05																			
Asn	0.14	3.32																		
Asp	0.05	0.05	403.07																	
Cys	0.05	0.05	295.12	0.05																
Gln	0.05	0.83	0.05	30.65	0.05															
Glu	0.06	0.05	0.05	167	0.05	0.97														
Gly	37.76	2.53	53.76	396.12	160.46	0.23	0.05													
His	0.05	0.81	8.34	8.47	0.05	1.22	0.05	0.05												
Ile	1.65	0.05	58.58	0.05	596.63	0.05	0.06	37.88	0.05											
Leu	0.14	1.74	88.55	4.7	300.17	6.34	1.76	13.44	2.56	236.33										
Lys	0.05	0.27	17.5	17.65	26.2	1.03	0.21	0.05	0.05	1.43	0.58									
Met	7.68	0.05	0.05	9.91	119.49	3.01	0.84	0.05	0.05	272.55	353.09	12.25								
Phe	0.05	3.75	110.8	0.05	166.79	0.05	0.05	0.05	0.05	80.64	495.72	1.09	299.79							
Pro	9.1	0.05	41.7	273.85	404.99	3.25	2.4	8.35	2.3	28.96	425.27	8.22	0.05	79.23						
Ser	16.37	0.32	67.2	60.39	822.97	0.27	0.05	30.35	0.81	0.05	72.51	0.88	39.56	222.56	114.19					
Thr	1.58	0.05	6.77	1.78	162.09	0.05	0.05	0.05	0.05	51.39	6.23	0.09	27.04	1.94	4.38	17.88				
Trp	0.05	73.14	549.54	76.57	1377.89	412.56	8.88	0.05	25.65	0.05	324.74	75.99	51.48	226.58	1519.22	22.18	0.05			
Tyr	0.05	10.03	728.29	0.05	844.43	61.11	1.15	0.05	235.7	34.25	51.44	5.68	0.05	1411.98	329.88	41.86	0.37	293.27		
Val	61.67	0.05	0.05	10	490.52	0.05	1.26	139.5	0.18	458.7	262.81	0.05	337.36	161.37	102.67	114.73	74.1	0.05	47.84	
	Ala	Arg	Asn	Asp	Cys	Gln	Glu	Gly	His	Ile	Leu	Lys	Met	Phe	Pro	Ser	Thr	Trp	Tyr	
mtFishR2morphArg	0.05																			
Asn	0.22	1.11																		
Asp	0.05	0.05	405.52																	
Cys	0.05	0.05	338.21	238.86																
Gln	0.05	0.69	1.68	33.89	1.64															
Glu	0.06	0.05	0.05	173.51	0.05	1.04														
Gly	39.46	2.58	52.12	413.84	111.33	0.1	0.05													
His	0.05	0.21	8.78	8.86	0.05	1.57	0.05	0.05												
Ile	1.56	0.05	59.69	0.05	686.45	0.05	0.05	41.85	0.05											
Leu	0.05	1.52	86.4	4.59	319.67	6.88	2.18	11.81	2.8	246.35										
Lys	0.05	0.22	17.94	18.58	29.62	1.15	0.2	0.05	0.05	1.33	0.55									
Met	8.26	0.05	0.05	7.1	313.06	2.95	0.88	0.05	0.05	278.61	360.51	12.39								
Phe	0.05	3.09	140.31	0.05	175.19	0.05	0.05	0.05	0.41	73.45	515.27	0.88	306.52							
Pro	9.48	0.05	44.9	280.55	493.24	18.16	2.44	8.86	2.71	36.33	430.49	10.78	0.05	82.55						
Ser	17.52	0.05	69.51	60.03	777.13	0.5	0.05	32.99	0.84	0.05	73.83	0.94	39.4	235.42	117.83					
Thr	1.64	0.05	7.19	1.74	147.29	0.05	0.05	0.05	0.05	53.23	6.15	0.09	27.8	2.47	4.14	20.09				
Trp	0.05	134.43	648.09	0.05	809.54	308.04	1.89	0.05	10.6	0.05	415.35	90.92	93.05	236.34	653	32.52	0.05			
Tyr	0.05	33	735.51	0.05	945.35	58.79	0.93	0.05	252.17	31.89	51.73	5.17	0.05	1450.11	339.31	44.42	0.18	447.02		
Val	63.09	0.05	0.05	0.05	687.94	0.05	1.1	150.73	0.18	474.65	278.25	0.14	329.49	160.26	108.9	107.16	75.5	0.05	36.83	
	Ala	Arg	Asn	Asp	Cys	Gln	Glu	Gly	His	Ile	Leu	Lys	Met	Phe	Pro	Ser	Thr	Trp	Tyr	
mtFishR3molArg	267.83																			
Asn	0	31.74																		
Asp	0.23	0	0.44																	
Cys	0	0	4.3	0																
Gln	0	0	3.28	0	0															
Glu	0	655.11	25.28	89.41	0	97.06														
Gly	2.25	51.46	0.04	0	0.58	0	4.86													
His	0	303.61	0	0	0	654.18	389.25	0.03												
Ile	113.87	1767.33	95.21	0	59.4	39.03	212.07	0.61	38.67											
Leu	152.06	310.14	0	0	57.38	522.43	0	0	389.77	0										
Lys	1.15	264.23	9.41	0	0	228.92	108.09	0	0	228.52	177.84									
Met	144.34	375.11	0	0	189.18	271.37	0	0.76	0	384.92	25.74	0								
Phe	20.42	0	0	0.86	79.84	0	0	0	0	419.94	640.96	0	0							
Pro	0.27	0.36	0	0	0	4.63	0	0	0	0	50.62	0	3.51	1.43						
Ser	6.41	3.17	0.19	0	15.93	0	6.73	0.07	0	33.75	12.05	0.2	0	0.93	0.04					
Thr	41.55	0	0	0.49	0	0	43.59	0	0	490.73	349.75	18.79	245.92	0	0.39	9.74				
Trp	483.41	0	0	0	0	0	0	91.57	0	0	0	0	0	0	0	17.91	0			
Tyr	3.91	0	159.34	7.49	0	77.27	517.31	1.33	865.74	0	91.15	188.14	0	315.37	0	8.82	142.98	515.14		
Val	296.7	0	0	0	163.98	267.19	33.42	0	0	29.26	985.75	0	285.79	477.9	0	93.57	274.72	632.38	720.44	
	Ala	Arg	Asn	Asp	Cys	Gln	Glu	Gly	His	Ile	Leu	Lys	Met	Phe	Pro	Ser	Thr	Trp	Tyr	
mtFishR3morphArg	256.94																			
Asn	0.15	5.83																		
Asp	0.17	0	0.52																	
Cys	0	0	4.27	0																
Gln	0	0	2.3	0	0															
Glu	0	702.91	22.48	72.75	0	79.01														
Gly	2.03	55.99	0.05	0	0.52	0	4.43													
His	5.77	190.42	9.24	0	0	583.42	389.72	0.18												
Ile	49.74	148.72	9.46	1.48	0	0	0	1.28	0											
Leu	130.18	205.98	0	0	55.02	287.29	13.06	0	304.82	277.4										
Lys	12.68	81.53	9.96	0	0	213.6	113.59	0	21.32	0	147.99									
Met	109.99	281.18	0	0	188.48	234.12	0	0.59	0	570.31	0	0								
Phe	28.7	0	0	0.83	129.11	0	0	0	0	0	565.99	0	0							
Pro	0.21	0.29	0	0	0	4.78	0	0	0	0.19	47.55	0	3.03	1.2						
Ser	8.4	4.99	0.2	0	15.75	0	6.12	0.06	0	2.73	2.93	0.09	0	0.72	0.05					
Thr	41.85	0	2.02	0.69	1.13	1.24	41.24	0	8.32	121.01	306.48	19.17	212.85	1.03	0.18	10				
Trp	463.03	0	0	0	0	0	0	81.6	0	0	0	0	0	0	0	14.42	0			
Tyr	55.79	3365.84	116.75	1.09	0	0	309.48	0.61	273.46	156.22	0	268.96	5.39	492.01	0	24.09	287.9	188.02		
Val	102.11	0	0	0.11	17.62	517.23	22.55	0	50.36	3.37	2216.88	0	529.79	528.37	0	73.51	235.55	0	1147.84	
	Ala	Arg	Asn	Asp	Cys	Gln	Glu	Gly	His	Ile	Leu	Lys	Met	Phe	Pro	Ser	Thr	Trp	Tyr	
mtFishR0molArg	0.18																			
Asn	30.48	48.98																		
Asp	13.57	0.18	884.56																	
Cys	11.3	0.18	70.08	0.18																
Gln	3.88	297.66	123.02	39.09	0.18															
Glu	25.68	4.42	98.99	781.45	0.18	366.47														
Gly	170.36	15.76	136.72	52.2	0.18	5	51.96													
His	19.26	196.93	311.51	99.17	77.19	574.68	28.57	3.1												
Ile	130.15	0.18	17.77	0.18	46.92	2.1	10.32	4.98	5.77											
Leu	31.24	15.29	7.29	0.18	18.37	36	3.27	2.67	16.05	395.5										
Lys	16.8	63.75	348.4	14.22	21.99	389.83	158.35	8.27	21.03	7.69	7.39									
Met	167.95	2.35	0.45	3.62	10.95	52.95	8.41	5.83	4.7	580.54	571.65	98.11								
Phe	21.92	1.56	12.83	0.18	115.59	7.19	0.18	1.71	33.63	103.27	255.16	2.13	58.73							
Pro	48.09	7.41	14.18	5.03	0.18	86.71	1.92	2.69	31.19	6.34	26.16	37.43	1.11	10.09						
Ser	496.37	15.84	558.97	130.01	398.04	67.88	29.37	146.25	59.76	13.95	39.77	54.8	71.85	59.6	154.42					
Thr	683.53	5.44	217.7	40.37	96.55	33.8	28.63	11.61	17.35	400.01	91.41	83.38	444.2	28.36	93.48	733.14				
Trp	0.18	13.97	0.18	0.18	84.32	18.88	3.57	6.05	10.08	0.18	20.21	13.82	9.32	15.4	9.87	17.37	0.48			
Tyr	1.14	8.41	113.64	14.17	182.25	49.29	0.18	4.76	507.64	18.17	25.83	20.8	9.05	536.4	14.09	58.45	22.27	47.85		
Val	229.34	2.81	11.2	11.51	0.18	11.28	6.48	8.63	1.42	1834.94	147.43	5.96	376.79	31.26	12.56	19.89	364.57	7.63	6.37	
	Ala	Arg	Asn	Asp	Cys	Gln	Glu	Gly	His	Ile	Leu	Lys	Met	Phe	Pro	Ser	Thr	Trp	Tyr	
mtFishR0morphArg	0.18																			
Asn	30.66	48.92																		
Asp	13.6	0.18	884.98																	
Cys	11.31	0.18	70.08	0.18																
Gln	3.95	296.19	125.84	33.99	0.18															
Glu	25.89	4.42	96.06	784.68	0.18	365.96														
Gly	170.82	15.75	137.04	51.9	0.18	5.89	51.97													
His	19.04	198.25	313	100.56	77.12	574.62	29.15	3.13												
Ile	130.3	1.21	17.89	0.18	47.03	2.09	10.28	4.94	5.76											
Leu	31.26	14.71	7.3	0.18	18.36	36.34	3.68	2.58	16.23	394.81										
Lys	17.86	64.05	348.24	14.27	22	396.72	158.37	8.23	13.29	6.32	7.47									
Met	168.28	2.38	0.18	3.92	11.19	52.95	8.27	5.81	4.32	581.58	570.32	98.02								
Phe	21.46	1.56	12.81	0.18	115.52	7.16	0.18	1.71	35.06	104.76	255.41	2.12	59.57							
Pro	47.97	7.41	12.71	5.06	0.18	86.9	1.76	2.69	31.23	6.01	26.07	38.42	1.11	10.09						
Ser	495.57	15.88	559.85	129	397.79	64.75	29.27	146.49	57.78	13.71	39.82	54.57	71.57	60.43	152.79					
Thr	686.89	5.46	221.64	40.14	96.52	34.72	29.04	11.12	17.75	398.97	92.01	82.11	444.19	28.2	94.44	733.18				
Trp	0.18	13.96	0.18	0.18	84.25	18.87	3.56	6.04	10.08	0.18	19.61	13.81	9.35	15.42	9.84	17.36	0.49			
Tyr	1.49	8.39	113.58	14.1	181.71	51.25	0.18	4.76	506.97	16.86	25.54	21.2	9.46	534.63	14.8	58.58	21.6	47.89		
Val	229.27	2.88	11.09	11.5	0.18	11.08	6.47	8.5	1.36	1835.3	147.13	6.1	373.38	31.34	12.06	19.52	366.31	7.65	6.46	
	Ala	Arg	Asn	Asp	Cys	Gln	Glu	Gly	His	Ile	Leu	Lys	Met	Phe	Pro	Ser	Thr	Trp	Tyr	
