# Supplementary material for: Improving Evolutionary Models for Mitochondrial Protein Data with Site-Class Specific Amino Acid Exchangeability Matrices
Source: PLoS One. 2013 Jan 31;8(1):e55816. doi: 10.1371/journal.pone.0055816 (PMC3561347; doi:10.1371/journal.pone.0055816)
Supplement: MethodsS1 — Sequence alignment methods. This supplemental methods section provides a detailed description of the protocol for sequence alignment. In addition, the method of post-alignment filtering of sites is described and a list of sites excluded from subsequent analysis is provided. (PDF) [file pone.0055816.s010.pdf]

MethodsS1 Sequence alignment methods

The complete mitochondrial genomes for 143 taxa were downloaded from GenBank. Using perl scripts that had been developed for genome data, all sequences were parsed out of the genomes into one flat file. Blastclust was used to cluster the data from the flat file. A sequence similarity index of 60% was used which was found to maximally cluster the genes. Ten of the genes (CO1, CO2, CO3, ND1, ND3, ND4, ND4L, ND5, cytochrome b and atpase 6) clustered all representatives. ND2 was lacking one taxon (*Erinaceus europaeus*, NC_002080), and the sequence for that gene from that taxon was added to the ND2 file. ATPase 8 was short by 19 taxa, those sequences were added to the ATPase 8 file. ND6 was not used in this study as it codes on the opposite strand to the rest of the genes. The 143 amino acid sequences for each gene were aligned using t-coffee (Notredame et al 2000) under the default settings. Using perl scripts amino acid alignments were used to create DNA alignments. Alignments were inspected by eye using GeneDoc software. Amino acids that were ambiguous (at the beginning and ends of sequences) and those amino acids, which overlapped between genes, were removed (the same sequence was removed for both the AA and nucleotide data). Amino acid sites removed (numbering based on human sequence, NC_012920) from aligned mitochondrial mammal sequences were: *ND2* (344-347), *CO1* (513), *CO2* (222-227), *ATPase* 8 (1, 54-68), *ATPase* 6 (1-16) *CO3* (261), *ND4L* (97, 98), *ND4* (1-3), *ND5* (1, 2, 602, 603) *cytochrome b* (379, 380). Finally the genes were concatenated together into one sequence, the order of concatenation was: ND1, ND2, CO1, CO2, A8, A6, CO3, ND3, ND4L, ND4, ND5, cytb.

The nucleotide sequences were used to determine phylogenetic relationships between these taxa using Paup* (Swofford 2003). Third positions were excluded from the analysis and gaps were treated as missing data. A NJ tree, using the distance criterion with HKY85 and rates following a gamma distribution, was generated. The same procedure was followed for the fish data.

Notredame C, Higgins DG, Heringa J (2000) T-Coffee: A novel method for fast and accurate multiple sequence alignment. J Mol Biol 302: 205-217.

Swofford DL (2003) PAUP* Phylogenetic analysis using parsimony (*and other methods). Version 4. Sinauer Associates, Sunderland, Massachusetts.
